# Supplementary material for: Careful design of Large Language Model pipelines enables expert-level retrieval of evidence-based information from syntheses and databases
Source: PLoS One. 2025 May 15;20(5):e0323563. doi: 10.1371/journal.pone.0323563 (PMC12080840; doi:10.1371/journal.pone.0323563)
Supplement: S1 File — (DOCX) [file pone.0323563.s001.docx]

File S1 – Participant Information Sheet and Consent Form

**Participant Information Sheet**

**Research Project:**Evaluating retrieval augmented language models for conservation decision making

**Funding and Sponsorship**
This research is funded by AI@Cam and sponsored by the University of Cambridge.

**Purpose of the Research**
The primary purpose of this experiment is to study question answering in the context of conservation decision making. We will compare the ability of human participants (both experts and non-experts) to navigate the Conservation Evidence (CE) database and answer
multiple-choice questions with the performance of Large Language Models (LLMs) on the same task. This information will be used to evaluate the LLMs' ability to retrieve correct information from the CE database and generate accurate answers based on the retrieved information.

**Participation Details**

If you choose to participate, you will be asked to:
● Answer 47 multiple-choice questions via an online survey
● Use the database available at www.conservationevidence.com to answer these questions
● Self-report the total time taken to answer all questions attempted

The estimated time commitment for all 47 is approximately one and a half hours but you are free to answer as many as you like.

**Data Collection**
No personal data will be collected as part of this study. All responses will be anonymous.

**Benefits of Participation**
While there are no direct benefits to participants, this research aims to develop and assess techniques for using Large Language Models to retrieve information from the Conservation Evidence database and offer advice in response to natural language questions. This has the
potential to broaden the use of data in Conservation Evidence, contributing to better conservation decision-making processes.

**Risks and Disadvantages**
There are no known risks associated with participation in this study. The only potential disadvantage is the time required to complete the task (estimated at ninety minutes).

**Voluntary Participation and Withdrawal**
Participation in this study is entirely voluntary. However, due to the anonymous nature of data collection, it will not be possible to withdraw your data once you have submitted your responses. Please consider this carefully before agreeing to participate. You are free to leave the survey at any time but your responses may be recorded.

**Data Handling and Protection**
All data collected will be anonymized and stored securely in the Microsoft cloud environment.

**Dissemination of Results**
The results from this research are intended for communication in an academic publication.

**Future Use of Data**
The anonymized data may be used for future research, particularly if new AI models become available that make reassessing effectiveness worthwhile.

**Contact Information**
For further information or to raise any concerns, please contact:
● Sadiq Jaffer (sj514@cam.ac.uk)
● Sam Reynolds (sar87@cam.ac.uk)

**Use of Personal Information**
For more information about how the University of Cambridge uses personal data for research purposes, please visit:
<https://www.information-compliance.admin.cam.ac.uk/data-protection/research-participant-data>

**Consent Form**

Please read the following statements carefully. By proceeding with the survey, you are
indicating that you have read, understood, and agree to these statements:

1. I confirm that I have read and understood the Participant Information Sheet for the
study "Evaluating retrieval augmented language models for conservation decision
making".

2. I have been given the opportunity to ask questions about the project and have had
these answered satisfactorily.

3. I understand that my participation is voluntary and that I am free to decline to answer
any particular question.

4. I understand that once I submit my responses, I will not be able to withdraw my data
from the study due to its anonymous nature.

5. I understand that my responses will be kept strictly confidential and that all data will
be anonymized.

6. I agree that the anonymized data collected from me can be used for future research
projects.

7. I agree to take part in the above study.

If you agree with all of the above statements and wish to participate in this study, please
proceed with the survey. By continuing, you are giving your consent to participate under the
conditions described above.

If you do not agree with these statements or do not wish to participate, please close this
browser window now.

For any questions or concerns, please contact:
● Sadiq Jaffer (sj514@cam.ac.uk)
● Sam Reynolds ([sar87@cam.ac.uk](mailto:sar87@cam.ac.uk))

*****IMPORTANT PLEASE READ*****

This survey will show you 45 questions in a randomised order. Each question is multiple choice and your role is to select the correct response based on consulting the Conservation Evidence database available at [www.conservationevidence.com](http://www.conservationevidence.com). Please do not use any other resource to answer the questions and we ask that you state the URL of the Conservation Evidence action page used (e.g., <https://conservationevidence.com/actions/2983>).
