# Supplementary material for: Careful design of Large Language Model pipelines enables expert-level retrieval of evidence-based information from syntheses and databases
Source: PLoS One. 2025 May 15;20(5):e0323563. doi: 10.1371/journal.pone.0323563 (PMC12080840; doi:10.1371/journal.pone.0323563)
Supplement: S2 File — (DOCX) [file pone.0323563.s002.docx]

Supporting Information for “Careful design of Large Language Model pipelines enables expert-level retrieval of evidence-based information from syntheses and databases”

# Information on structure of Conservation Evidence database summaries

Figure S1 – Example of key messages from similar action pages (source documents) overlapping for a question. The source action document for the question is on the left (Leave headlands in fields unsprayed (conservation headlands)) but the action on the right was also used by human experts and LLMs to get the correct answer to the question.

**Action: Leave headlands in fields unsprayed (conservation headlands).** Key messages: Twenty-two studies from 14 replicated, controlled experiments (of which two randomized) including two reviews, from a total of 32 studies from 20 experiments (of which 17 replicated, controlled) including three reviews from Finland, Germany, the Netherlands, Sweden and the UK that investigated species richness and diversity of farmland wildlife found that conservation headlands contained higher species richness or diversity of invertebrates or plants than other habitat types. Twelve studies (including a review) from ten replicated experiments (of which eight controlled and three controlled and randomized) found that some or all invertebrates or plants investigated did not have higher species richness or diversity on conservation headlands compared to other habitat types. This included both replicated, controlled studies investigating bee diversity. Two replicated studies from the UK found that unfertilized conservation headlands had more plant species than fertilized conservation headlands.

Positive effects of conservation headlands on abundances or behaviours of some or all species investigated were found by 27 studies from 15 replicated experiments (of which 13 controlled) including five reviews out of a total of 36 studies from 20 experiments (17 replicated, controlled) including five reviews from Finland, Germany, the Netherlands, Sweden and the UK that investigated birds (some studies looked at number of visits), mammals (some studies looked at number of visits), invertebrates and plant abundance/cover. One review from the UK found a positive effect on grey partridge populations but did not separate the effects of several other interventions including conservation headlands. Nineteen studies from 13 replicated (12 controlled) experiments and a review from Finland, Germany, the Netherlands and the UK found that some or all species of birds, invertebrates or plants investigated were at similar, or lower, abundances on conservation headlands compared to other management. One review from the UK and a study in Germany found conservation headlands had a positive effect on plants and some, but not all invertebrates, or rare arable weeds but did not specify how.

All eight studies from the UK and Sweden that investigated species’ productivity, from three replicated (two controlled) experiments including two reviews found that grey partridge productivity or survival was higher in conservation headlands (or in sites with conservation headlands), compared to other management. One replicated study from the UK found that conservation headlands did not increase the proportion of young grey partridges in the population. A before-and-after study from the UK found that some invertebrates in conservation headlands survived pesticide applications to neighbouring fields. A review found crop margins reduce the effects of spray drift on butterflies.

A replicated study from Germany and a review found that conservation headlands appeared to prevent or reduce the establishment and spread of pernicious weeds.

**Action: Reduce fertilizer, pesticide or herbicide use generally.**

Key messages: Of 38 individual studies from Austria, the Czech Republic, Denmark, Finland, France, Germany, Ireland, the Netherlands, Sweden and the UK investigating the effects of reducing fertilizers, pesticides or herbicides, 34 studies (23 replicated, of which six also controlled and randomized, one review and one systematic review) found benefits to some invertebrates, plants, or farmland birds. Twenty-five studies (16 replicated, of which seven also randomized and controlled and one review) found negative, mixed, minimal or no effects on some invertebrates, farmland birds or plants.

Ten studies (six replicated, controlled studies of which two randomized) from three countries found positive effects of reducing or stopping pesticide applications on invertebrates, plants, or birds. Eight studies (two replicated controlled and randomized, one paired before-and-after trial) from four countries found inconsistent or no effects on some invertebrates or birds.

Ten studies (nine replicated, five also controlled and a European systematic review) from four countries found positive effects of reducing or stopping herbicide use on plants, invertebrates, and birds. Five replicated studies (two also controlled and randomized) from three countries found no or mixed effects on birds, invertebrates and plants.

Five studies (three replicated controlled of which two randomized) from four countries found positive effects of reducing or stopping fertilizer applications on invertebrates, Eurasian skylark, or plants. Four studies (three replicated, controlled and randomized) from two countries found reducing or stopping fertilizer inputs had no, or no consistent effects on some invertebrates and farmland birds. Two studies from the UK (one replicated) found plots where fertilizer inputs were not reduced tended to have higher earthworm biomass or abundance.

Fifteen studies (three replicated controlled of which one also randomized, five site comparisons and one review) from seven countries looked at the effects of reducing or stopping applications of two or more inputs: pesticides, herbicides, or fertilizers. Thirteen studies found positive effects of reducing two or more inputs on some or all invertebrates, plants, soil organisms, and birds studied. Seven studies found negative or no effects of reducing combinations of inputs on some invertebrates, plants or birds.

**Question:** Which of the following factors is most likely to enhance the effectiveness of conservation headlands for plant diversity?

**Synopsis:** Farmland Conservation

**Key messages**

- **Two studies**evaluated the effects of prohibiting activities that cause disturbance in sensitive areas for marine mammals. One study was in the [Kattegat Sea](https://conservationevidence.com/actions/2917) (Denmark) and one in the [Indian Ocean](https://conservationevidence.com/actions/2917) (Australia).

COMMUNITY RESPONSE (0 STUDIES)

**POPULATION RESPONSE (1 STUDY)**

- **Abundance (1 study):**[One before-and-after study](https://conservationevidence.com/actions/2917) in the Kattegat sea found that harbour porpoise activity increased at a stony reef after fishing was prohibited and the reef was restored with boulders.

**BEHAVIOUR (1 STUDY)**

- **Behaviour change (1 study):**[One site comparison study](https://conservationevidence.com/actions/2917) in the Indian Ocean found that a beach where human access was fully prohibited had fewer Australian sea lions showing aggression or retreating compared to a beach where access was partly prohibited.

**Supporting evidence from individual studies:**

1. A before-and-after study in 2006–2012 of a stony reef in the Kattegat sea, Denmark (Mikkelsen *et al.* 2013) found that prohibiting fishing, along with restoring the reef, resulted in harbour porpoise *Phocoena phocoena* echolocation clicks being recorded more often and for longer periods than before protection and restoration. The average number of minutes with porpoise recordings and the average duration of porpoise encounters were higher at the reef in each of four years after fishing was prohibited and the reef restored (13–15 minutes/day; 4–5 minutes) than during two years before (6–10 minutes/day; 3 minutes). Porpoise activity at an intact reef 10 km away decreased over the same period (‘before’: 11–15 minutes/day; ‘after’: 3–7 minutes/day). In June–September 2008, a total of 100,000 t of norite boulders were dumped over 19 days to restore a 45,000 m^2^ cavernous stony reef. Fishing was prohibited around the restored reef in 2009–2012. Porpoise activity was recorded with acoustic data loggers (two at the restored reef; two at an intact reef) for 33–75 days in June–August in each of two years before protection and restoration (2006 and 2007) and each of four years after (2009–2012).

[Study and other actions tested](https://conservationevidence.com/individual-study/8777)

Referenced paper:

[Mikkelsen L., Mouritsen K.N., Dahl K., Teilmann J. & Tougaard J. (2013) Re-established stony reef attracts harbour porpoises *Phocoena phocoena*. *Marine Ecology Progress Series*, 481, 239-248.](https://doi.org/10.3354/meps10260)

1. A site comparison study in 2013–2014 of two beaches on islands in the Indian Ocean, Western Australia (Osterrieder *et al.* 2017) found that a beach where human access was prohibited had fewer Australian sea lions *Neophoca cinerea*showing aggression or retreating compared to a beach where access was partly prohibited. The number of responses to vessels and people in which sea lions showed aggression (gaping or lunging) or retreated were lower at a beach where access was fully prohibited (aggression: 0 responses/h; retreated: 3 responses/h) than at a beach where access was partly prohibited (aggression: 14 responses/h; retreated: 21 responses/h). Less severe responses (e.g. sitting upright, lifting head, looking or entering the water) did not differ significantly between the two beaches (see original paper for data). Both beaches were sea lion haul-out sites designated as sanctuary zones. At one site, public access to the beach was fully prohibited, with viewing permitted from the water only via kayak or boat tours. At the other site, public access was permitted to part of the beach and prohibited in all other areas. Individual responses of sea lions to vessels and people were recorded by observers or remote live video cameras overlooking each of the two sites for a total of 134–142 h during 19–20 days in November–April 2013/2014.

[Study and other actions tested](https://conservationevidence.com/individual-study/8728)

Referenced paper:

[Osterrieder S.K., Salgado Kent C. & Robinson R.W. (2017) Responses of Australian sea lions, *Neophoca cinerea*, to anthropogenic activities in the Perth metropolitan area, Western Australia. *Aquatic Conservation: Marine and Freshwater Ecosystems*, 27, 414-435.](https://onlinelibrary.wiley.com/doi/epdf/10.1002/aqc.2668)

**Background information and definitions:**

There are many human activities that may cause disturbance and harm to marine and freshwater mammals and their habitats. This intervention involves prohibiting any such activity that may cause disturbance in particularly sensitive areas for marine and freshwater mammals. For example, in important feeding or resting grounds.

**For studies that involve prohibiting activities as part of legal protection of an area, see**[***Legally protect habitat for marine and freshwater mammals***](https://conservationevidence.com/actions/2915)***.***

**Synopsis:** Marine and Freshwater Mammal Conservation

**Action:** Cease or prohibit activities that cause disturbance in sensitive areas for marine and freshwater mammals

**Overall effectiveness category:** Likely to be beneficial

**Number of studies:** 2

Figure S2 – Example of evidence summaries for a Conservation Evidence action page. This one can be found at: <https://conservationevidence.com/actions/2917>.

# Prompt structure for exam generation and evaluations

Table S1 – Prompt provided to Claude 3.5 Sonnet to generate initial unfiltered exam questions and prompts given to LLMs depending on the exam condition. We include some example prompts used for certain questions.

| Exam generation or condition | Prompt structure | Example |
| --- | --- | --- |
| Exam generation | See example for prompt used to generate all unfiltered questions. | Generate 3-4 difficult multi-form exam questions based on the provided conservation action information.  Each question should have 4 options, of which 1 is the correct answer (the correct answer should not always be the longest answer), and explanations. Follow these guidelines:  1. Include a mix of question types. These may include:  a) Effects/outcomes of a specific conservation action  b) Actions to achieve specific conservation objectives/outcomes  c) Alternatives to achieve a particular conservation goal  d) Trade-offs between different conservation actions  e) Geographical and contextual variations in action effectiveness  f) Scaling of actions and their impacts  g) Timeframes for expected outcomes  h) Factors associated with success or failure of conservation efforts  2. Include questions about achieving desired outcomes (e.g., "How to increase the abundance of native bees?"). Pay attention to but do not explicitly ask questions about the effectiveness rating.  3. Focus on conservation actions, outcomes, and their relationships. With actions referencing more than one study, see how they compare or differ.  4. Ask questions about the context provided, but DO NOT reference specific studies or use past tense (e.g. do not have questions like "Based on the information, provided...").  5. Use Bloom's taxonomy (Remember, Create, Evaluate, Analyse, Apply, Understand) for question variety. Use these and only these category names.  6. Provide documentation from the text itself (source) and a proof of correctness that includes proof that the incorrect answers are not supported by the rest of the document.  7. Ensure that only one candidate answer is the correct one.  Evaluate your assignment of Bloom's category AFTER generating each question.  Format each question as a JSON object:  {{  "question": "...",  "source_action": {action_number},  "documentation": "...",  "A": "...",  "B": "...",  "C": "...",  "D": "...",  "correct_answer": "...",  "proof_of_correctness": "...",  "bloom_level": "..."  }}  Ensure that once you have generated these questions, you recheck them for clarity, correctness and difficulty (these should not have necessarily obvious answers - use the source material). You may remove questions as you deem appropriate, but you must return at least ONE.  Output these questions as a valid JSON array of question objects, starting with '[' and ending with ']'. |
| Closed book | Question. | You are given a multiple choice question with 4 options.  You must give an answer for the question - return only one character as output, nothing else, ensure it is a capital letter A, B, C or D.    Question: In the context of bumblebee conservation, which of the following strategies would likely be most effective for increasing the density of nest-searching queen bumblebees in agricultural landscapes?    A: Establishing grassy field margins around crop fields  B: Planting more hedgerows along field boundaries  C: Expanding the area of unmanaged grasslands  D: Increasing the area of managed grasslands |
| Oracle | Question. [Source document text]. | You are given a multiple choice question with 4 options. You are given retrieved context from the Conservation Evidence database. This database contains studies and actions related to conservation efforts. You must give an answer to the question - return only one character as output, nothing else, and ensure it is a capital letter A, B, C or D.  Question: In the context of bumblebee conservation, which of the following strategies would likely be most effective for increasing the density of nest-searching queen bumblebees in agricultural landscapes?    Conservation Evidence database information: Action Name:12: Increase areas of rough grassland for bumblebee nesting  Effectiveness rating: Awaiting assessment  Key Messages:  One replicated controlled trial on lowland farms in Scotland showed that grassy field margins attracted nest-searching queen bumblebees in spring at higher densities than cropped field margins, managed or unmanaged grasslands or hedgerows.    Synopsis Details:  Title: Bee Conservation  Description: Bee Synopsis  A: Establishing grassy field margins around crop fields  B: Planting more hedgerows along field boundaries  C: Expanding the area of unmanaged grasslands  D: Increasing the area of managed grasslands |
| Hybrid, sparse, dense retrieval | Question. [Document 1 text]. [Document 2 text]. | You are given a multiple choice question with 4 options. You are given retrieved context from the Conservation Evidence database. This database contains studies and actions related to conservation efforts. You must give an answer to the question - return only one character as output, nothing else, and ensure it is a capital letter A, B, C or D.  Question: In the context of bumblebee conservation, which of the following strategies would likely be most effective for increasing the density of nest-searching queen bumblebees in agricultural landscapes?  Conservation Evidence database information:  Action Name 1: 32: Manage wild honey bees sustainably Effectiveness rating: Awaiting assessment Key Messages: We can find no evidence of the impact of reduced honey-hunting or improved harvesting methods on wild honey bee populations. One trial in southern Vietnam, showed that occupancy of artificial rafters by the giant honey bee Apis dorsata can be over 85% when rafters are placed by a large clearing greater than 25 m in diameter.  Synopsis Details: Title: Bee Conservation Description: Bee Synopsis  Action Name 2: 12: Increase areas of rough grassland for bumblebee nesting Effectiveness rating: Awaiting assessment Key Messages: One replicated controlled trial on lowland farms in Scotland showed that grassy field margins attracted nest-searching queen bumblebees in spring at higher densities than cropped field margins, managed or unmanaged grasslands or hedgerows.  Synopsis Details: Title: Bee Conservation Description: Bee Synopsis A: Establishing grassy field margins around crop fields  B: Planting more hedgerows along field boundaries  C: Expanding the area of unmanaged grasslands  D: Increasing the area of managed grasslands |
| Confused | Question. [Source document text]. [Confusion document text]. |  |

# Comparison of LLM performance against random guesser

Table S2 – Results of a paired comparison of Open Book (Hybrid retrieval) Large Language Model and retrieval strategies versus a random guesser on the filtered 45-question dataset. Test statistics are from a permutation test used to test the null hypothesis: there is no difference in accuracy of the given LLM and a random guesser (with a 25% chance of correctly answering a question).

| **Large Language Model** | **Overall percentage accuracy** | **Holm- adjusted p-value** | **Mean permutation test statistic** | **SD permutation test statistic** | **Lower 95% CI** | **Upper 95% CI** |
| --- | --- | --- | --- | --- | --- | --- |
| GPT-4o | 97.80% | 0 | 32.765 | 2.911 | 27 | 38 |
| Llama 3.1 70B Instruct Turbo | 97.80% | 0 | 32.754 | 2.908 | 27 | 38 |
| Gemma2 - 27B Instruct | 95.60% | 0 | 31.732 | 2.880 | 26 | 37 |
| GPT-4o Mini | 93.30% | 0 | 30.768 | 2.889 | 25 | 36 |
| Mixtral 8x22B | 93.30% | 0 | 30.752 | 2.904 | 25 | 36 |
| Claude 3.5 Sonnet | 93.30% | 0 | 30.734 | 2.912 | 25 | 36 |
| Gemini 1.5 Flash | 91.10% | 0 | 29.758 | 2.926 | 24 | 35 |
| Gemini 1.5 Pro | 91.10% | 0 | 29.732 | 2.930 | 24 | 35 |
| Gemma2 - 9B Instruct | 88.90% | 0 | 28.729 | 2.888 | 23 | 34 |
| Llama 3.1 8B Instruct Turbo | 86.70% | 0 | 27.768 | 2.895 | 22 | 33 |

# LLM accuracy across unfiltered questions

Table S3 – Analysis of deviance test to test statistical significance of explanatory categorical variables LLM, Exam type and Synopsis in a logistic regression with LLM accuracy as the response variable (Table S4, see Methods).

|  | LR Chisq | Df | Pr(>Chisq) |
| --- | --- | --- | --- |
| Large Language Model | 407.8226 | 9 | 0.0000 |
| Synopsis | 965.6664 | 23 | 0.0000 |
| Exam type | 6423.34 | 5 | 0.0000 |

Table S4 – Model summary for logistic regression of LLM accuracy (LLM accuracy ~ LLM + Synopsis + Exam type; see Methods).

|  | Estimate | Std. Error | z value | Pr(>\|z\|) |
| --- | --- | --- | --- | --- |
| (Intercept) | 0.990528883 | 0.05057618 | 19.58488907 | 0.0000 |
| Model_gemini_flash | 0.061948563 | 0.036505177 | 1.696980239 | 0.089700397 |
| Model_gemini_pro001 | 0.177655795 | 0.037157718 | 4.781127658 | 0.0000 |
| Model_gemma_27b | 0.137489675 | 0.036923506 | 3.72363537 | 0.000196374 |
| Model_gemma_9b | 0.010504471 | 0.036236296 | 0.289888108 | 0.77190184 |
| Model_gpt_4o | 0.353944426 | 0.038288375 | 9.244174707 | 0.0000 |
| Model_gpt_4o_mini | 0.106592174 | 0.036748957 | 2.900549648 | 0.003725088 |
| Model_llama_31_70b | 0.242826349 | 0.03755579 | 6.465750081 | 0.0000 |
| Model_llama_31_8b | -0.25135898 | 0.03505356 | -7.170711929 | 0.0000 |
| Model_mixtral_822 | -0.11479508 | 0.035632952 | -3.221598946 | 0.001274774 |
| Synopsis_Bat Conservation | 0.096808703 | 0.075128879 | 1.288568439 | 0.197548162 |
| Synopsis_Bee Conservation | 0.150895174 | 0.081448878 | 1.852636605 | 0.063934462 |
| Synopsis_Biodiversity of Marine Artificial Structures | 0.447152376 | 0.091366995 | 4.894025187 | 0.0000 |
| Synopsis_Bird Conservation | -0.54712378 | 0.046667378 | -11.72390237 | 0.0000 |
| Synopsis_Butterfly and Moth Conservation | -0.616705696 | 0.052878714 | -11.66264556 | 0.0000 |
| Synopsis_Control of Freshwater Invasive Species | -0.68742205 | 0.063202285 | -10.87653785 | 0.0000 |
| Synopsis_Farmland Conservation | -0.015154874 | 0.063605539 | -0.23826344 | 0.811676775 |
| Synopsis_Forest Conservation | -0.572801689 | 0.064256152 | -8.914347856 | 0.0000 |
| Synopsis_Grassland Conservation | -0.420604545 | 0.077569076 | -5.422322469 | 0.0000 |
| Synopsis_Management of Captive Animals | -0.212181915 | 0.077615154 | -2.733769179 | 0.006261392 |
| Synopsis_Marine and Freshwater Mammal Conservation | -0.283161953 | 0.072967494 | -3.880658879 | 0.000104174 |
| Synopsis_Marine Fish Conservation | -0.599273228 | 0.058486747 | -10.24630815 | 0.0000 |
| Synopsis_Marsh and Swamp Conservation | -0.395184531 | 0.050189589 | -7.87383475 | 0.0000 |
| Synopsis_Mediterranean Farmland | -0.242342746 | 0.065397086 | -3.705711693 | 0.000210798 |
| Synopsis_Natural Pest Control | 0.577817686 | 0.136811077 | 4.22347151 | 0.0000 |
| Synopsis_Peatland Conservation | -0.594128908 | 0.060491715 | -9.821657535 | 0.0000 |
| Synopsis_Primate Conservation | -0.520817358 | 0.062471984 | -8.336814758 | 0.0000 |
| Synopsis_Reptile Conservation | -0.762447063 | 0.049909633 | -15.27655124 | 0.0000 |
| Synopsis_Shrubland and Heathland Conservation | -0.55172218 | 0.067356704 | -8.191050754 | 0.0000 |
| Synopsis_Soil Fertility | -0.22498996 | 0.096379781 | -2.334410356 | 0.019574245 |
| Synopsis_Subtidal Benthic Invertebrate Conservation | -0.253135497 | 0.05981813 | -4.231752059 | 0.0000 |
| Synopsis_Sustainable Aquaculture | -0.029484627 | 0.103851848 | -0.283910473 | 0.77647899 |
| Synopsis_Terrestrial Mammal Conservation | -0.40875285 | 0.049872638 | -8.195933957 | 0.0000 |
| Category_confused | 1.741593825 | 0.03051498 | 57.07340625 | 0 |
| Category_dense_retrieval | 0.915710888 | 0.024863022 | 36.8302321 | 0.0000 |
| Category_hybrid_retrieval | 1.174193249 | 0.026250783 | 44.72983722 | 0 |
| Category_oracle | 1.988570505 | 0.032998497 | 60.26245711 | 0 |
| Category_sparse_retrieval | 0.72147999 | 0.024010844 | 30.04808951 | 0.0000 |

Table S5 – Estimated Marginal Means comparisons of LLM accuracy between synopses for the unfiltered dataset based on the logistic regression in Table S4.

| contrast | odds.ratio | SE | z.ratio (red = negative, green = positive) | p.value (red p<0.05) | abs.z.ratio |
| --- | --- | --- | --- | --- | --- |
| Amphibian Conservation / Reptile Conservation | 2.143515125 | 0.106982053 | 15.27655124 | 0.0000 | 15.27655 |
| Biodiversity of Marine Artificial Structures / Reptile Conservation | 3.352141648 | 0.28478989 | 14.23768468 | 0.0000 | 14.23768 |
| Farmland Conservation / Reptile Conservation | 2.111275335 | 0.113973938 | 13.8429854 | 0.0000 | 13.84299 |
| Bat Conservation / Reptile Conservation | 2.361402604 | 0.158640796 | 12.79020819 | 0.0000 | 12.79021 |
| Bee Conservation / Reptile Conservation | 2.492639619 | 0.184909301 | 12.3121608 | 0.0000 | 12.31216 |
| Biodiversity of Marine Artificial Structures / Butterfly and Moth Conservation | 2.897528326 | 0.251312869 | 12.26582191 | 0.0000 | 12.26582 |
| Biodiversity of Marine Artificial Structures / Control of Freshwater Invasive Species | 3.109849791 | 0.290420805 | 12.14911598 | 0.0000 | 12.14912 |
| Biodiversity of Marine Artificial Structures / Bird Conservation | 2.702767252 | 0.224578094 | 11.96598021 | 0.0000 | 11.96598 |
| Amphibian Conservation / Bird Conservation | 1.728274963 | 0.08065406 | 11.72390237 | 0.0000 | 11.7239 |
| Amphibian Conservation / Butterfly and Moth Conservation | 1.852814244 | 0.097974434 | 11.66264556 | 0.0000 | 11.66265 |
| Biodiversity of Marine Artificial Structures / Marine Fish Conservation | 2.847454976 | 0.257016767 | 11.59321171 | 0.0000 | 11.59321 |
| Biodiversity of Marine Artificial Structures / Peatland Conservation | 2.83284437 | 0.259414214 | 11.3709568 | 0.0000 | 11.37096 |
| Amphibian Conservation / Control of Freshwater Invasive Species | 1.988582454 | 0.125682956 | 10.87653785 | 0.0000 | 10.87654 |
| Biodiversity of Marine Artificial Structures / Forest Conservation | 2.773067381 | 0.260953164 | 10.83873177 | 0.0000 | 10.83873 |
| Butterfly and Moth Conservation / Farmland Conservation | 0.547961187 | 0.031091238 | -10.60190974 | 0.0000 | 10.60191 |
| Bird Conservation / Farmland Conservation | 0.587447202 | 0.029960394 | -10.43055862 | 0.0000 | 10.43056 |
| Biodiversity of Marine Artificial Structures / Primate Conservation | 2.632594165 | 0.244549768 | 10.42025717 | 0.0000 | 10.42026 |
| Biodiversity of Marine Artificial Structures / Shrubland and Heathland Conservation | 2.715224277 | 0.26133036 | 10.37831365 | 0.0000 | 10.37831 |
| Reptile Conservation / Subtidal Benthic Invertebrate Conservation | 0.600909122 | 0.029718713 | -10.29822418 | 0.0000 | 10.29822 |
| Bat Conservation / Butterfly and Moth Conservation | 2.041152091 | 0.141685564 | 10.27903878 | 0.0000 | 10.27904 |
| Amphibian Conservation / Marine Fish Conservation | 1.820795017 | 0.106492377 | 10.24630815 | 0.0000 | 10.24631 |
| Control of Freshwater Invasive Species / Farmland Conservation | 0.510549759 | 0.033934382 | -10.11439791 | 0.0000 | 10.1144 |
| Bat Conservation / Control of Freshwater Invasive Species | 2.190721086 | 0.169929589 | 10.11025128 | 0.0000 | 10.11025 |
| Natural Pest Control / Reptile Conservation | 3.820054729 | 0.506606639 | 10.10623292 | 0.0000 | 10.10623 |
| Biodiversity of Marine Artificial Structures / Terrestrial Mammal Conservation | 2.353503871 | 0.199888017 | 10.07752385 | 0.0000 | 10.07752 |
| Bee Conservation / Butterfly and Moth Conservation | 2.154590904 | 0.164202909 | 10.07208619 | 0.0000 | 10.07209 |
| Bee Conservation / Control of Freshwater Invasive Species | 2.312472327 | 0.193565012 | 10.0151642 | 0.0000 | 10.01516 |
| Bat Conservation / Bird Conservation | 1.903953441 | 0.123390711 | 9.936059695 | 0.0000 | 9.93606 |
| Biodiversity of Marine Artificial Structures / Marsh and Swamp Conservation | 2.321786442 | 0.197626896 | 9.896053874 | 0.0000 | 9.896054 |
| Marsh and Swamp Conservation / Reptile Conservation | 1.443776907 | 0.053753409 | 9.86440069 | 0.0000 | 9.864401 |
| Amphibian Conservation / Peatland Conservation | 1.811452316 | 0.109577857 | 9.821657535 | 0.0000 | 9.821658 |
| Bee Conservation / Bird Conservation | 2.009767319 | 0.144782944 | 9.689371185 | 0.0000 | 9.689371 |
| Reptile Conservation / Terrestrial Mammal Conservation | 0.702089624 | 0.025838287 | -9.610739105 | 0.0000 | 9.610739 |
| Bat Conservation / Marine Fish Conservation | 2.005878121 | 0.147985785 | 9.435065079 | 0.0000 | 9.435065 |
| Farmland Conservation / Marine Fish Conservation | 1.793409136 | 0.111190695 | 9.421320686 | 0.0000 | 9.421321 |
| Bee Conservation / Marine Fish Conservation | 2.117356553 | 0.169819416 | 9.353312024 | 0.0000 | 9.353312 |
| Mediterranean Farmland / Reptile Conservation | 1.682203124 | 0.094331425 | 9.274969713 | 0.0000 | 9.27497 |
| Bat Conservation / Peatland Conservation | 1.995585739 | 0.150418099 | 9.166617921 | 0.0000 | 9.166618 |
| Control of Freshwater Invasive Species / Natural Pest Control | 0.282171641 | 0.038987523 | -9.157154528 | 0.0000 | 9.157155 |
| Bee Conservation / Peatland Conservation | 2.106492163 | 0.172052146 | 9.121579877 | 0.0000 | 9.12158 |
| Farmland Conservation / Peatland Conservation | 1.784206955 | 0.114000939 | 9.061412157 | 0.0000 | 9.061412 |
| Butterfly and Moth Conservation / Natural Pest Control | 0.302848263 | 0.040509604 | -8.930211418 | 0.0000 | 8.930211 |
| Amphibian Conservation / Forest Conservation | 1.773228133 | 0.113940816 | 8.914347856 | 0.0000 | 8.914348 |
| Marine Fish Conservation / Natural Pest Control | 0.30817394 | 0.041935201 | -8.650220638 | 0.0000 | 8.650221 |
| Bee Conservation / Forest Conservation | 2.062042225 | 0.17424852 | 8.56416736 | 0.0000 | 8.564167 |
| Bird Conservation / Natural Pest Control | 0.324671471 | 0.042671535 | -8.55925145 | 0.0000 | 8.559251 |
| Natural Pest Control / Peatland Conservation | 3.22827066 | 0.442111921 | 8.557472953 | 0.0000 | 8.557473 |
| Bat Conservation / Forest Conservation | 1.953476082 | 0.15320844 | 8.537831744 | 0.0000 | 8.537832 |
| Biodiversity of Marine Artificial Structures / Grassland Conservation | 2.381562825 | 0.246847149 | 8.372053835 | 0.0000 | 8.372054 |
| Amphibian Conservation / Primate Conservation | 1.683403031 | 0.105165527 | 8.336814758 | 0.0000 | 8.336815 |
| Forest Conservation / Natural Pest Control | 0.316440713 | 0.043875714 | -8.298504716 | 0.0000 | 8.298505 |
| Farmland Conservation / Forest Conservation | 1.746557687 | 0.117839057 | 8.265191142 | 0.0000 | 8.265191 |
| Amphibian Conservation / Terrestrial Mammal Conservation | 1.504939729 | 0.075055315 | 8.195933957 | 0.0000 | 8.195934 |
| Amphibian Conservation / Shrubland and Heathland Conservation | 1.736240564 | 0.116947442 | 8.191050754 | 0.0000 | 8.191051 |
| Bee Conservation / Shrubland and Heathland Conservation | 2.019030315 | 0.175421045 | 8.086861756 | 0.0000 | 8.086862 |
| Bee Conservation / Primate Conservation | 1.957586882 | 0.162781314 | 8.077927421 | 0.0000 | 8.077927 |
| Natural Pest Control / Shrubland and Heathland Conservation | 3.094232413 | 0.433556038 | 8.061377448 | 0.0000 | 8.061377 |
| Bat Conservation / Primate Conservation | 1.854520294 | 0.142748949 | 8.0238774 | 0.0000 | 8.023877 |
| Bat Conservation / Shrubland and Heathland Conservation | 1.912728742 | 0.154908739 | 8.007706137 | 0.0000 | 8.007706 |
| Natural Pest Control / Primate Conservation | 3.000068268 | 0.413516316 | 7.970616896 | 0.0000 | 7.970617 |
| Amphibian Conservation / Marsh and Swamp Conservation | 1.484658131 | 0.074514382 | 7.87383475 | 0.0000 | 7.873835 |
| Management of Captive Animals / Reptile Conservation | 1.733712648 | 0.121260479 | 7.867374894 | 0.0000 | 7.867375 |
| Farmland Conservation / Primate Conservation | 1.65808361 | 0.109056135 | 7.688065198 | 0.0000 | 7.688065 |
| Biodiversity of Marine Artificial Structures / Subtidal Benthic Invertebrate Conservation | 2.014332496 | 0.183560143 | 7.684743534 | 0.0000 | 7.684744 |
| Farmland Conservation / Shrubland and Heathland Conservation | 1.710126434 | 0.120441753 | 7.618603271 | 0.0000 | 7.618603 |
| Bee Conservation / Terrestrial Mammal Conservation | 1.750056414 | 0.12977655 | 7.546938306 | 0.0000 | 7.546938 |
| Bat Conservation / Terrestrial Mammal Conservation | 1.657916267 | 0.111332586 | 7.528601947 | 0.0000 | 7.528602 |
| Reptile Conservation / Sustainable Aquaculture | 0.480483478 | 0.04720908 | -7.459928012 | 0.0000 | 7.459928 |
| Natural Pest Control / Terrestrial Mammal Conservation | 2.682020789 | 0.355637915 | 7.440159146 | 0.0000 | 7.440159 |
| Marine and Freshwater Mammal Conservation / Reptile Conservation | 1.6149195 | 0.1045572 | 7.402712286 | 0.0000 | 7.402712 |
| Bee Conservation / Marsh and Swamp Conservation | 1.726471456 | 0.128396151 | 7.342829339 | 0.0000 | 7.342829 |
| Marsh and Swamp Conservation / Natural Pest Control | 0.377946655 | 0.05016114 | -7.331231639 | 0.0000 | 7.331232 |
| Bat Conservation / Marsh and Swamp Conservation | 1.635573052 | 0.110217675 | 7.30092408 | 0.0000 | 7.300924 |
| Farmland Conservation / Terrestrial Mammal Conservation | 1.482304507 | 0.079969568 | 7.295675917 | 0.0000 | 7.295676 |
| Biodiversity of Marine Artificial Structures / Marine and Freshwater Mammal Conservation | 2.075732969 | 0.208093964 | 7.284870269 | 0.0000 | 7.28487 |
| Biodiversity of Marine Artificial Structures / Mediterranean Farmland | 1.992709204 | 0.189073235 | 7.266831139 | 0.0000 | 7.266831 |
| Farmland Conservation / Marsh and Swamp Conservation | 1.462327957 | 0.079320514 | 7.00610679 | 0.0000 | 7.006107 |
| Butterfly and Moth Conservation / Subtidal Benthic Invertebrate Conservation | 0.695189924 | 0.036464685 | -6.931373229 | 0.0000 | 6.931373 |
| Control of Freshwater Invasive Species / Subtidal Benthic Invertebrate Conservation | 0.647726621 | 0.040706815 | -6.910365261 | 0.0000 | 6.910365 |
| Grassland Conservation / Natural Pest Control | 0.368460328 | 0.053538008 | -6.871360991 | 0.0000 | 6.871361 |
| Bird Conservation / Reptile Conservation | 1.240262789 | 0.040081259 | 6.66290082 | 0.0000 | 6.662901 |
| Control of Freshwater Invasive Species / Mediterranean Farmland | 0.640773458 | 0.043686393 | -6.528234168 | 0.0000 | 6.528234 |
| Butterfly and Moth Conservation / Mediterranean Farmland | 0.687727256 | 0.040394256 | -6.373668732 | 0.0000 | 6.373669 |
| Bird Conservation / Subtidal Benthic Invertebrate Conservation | 0.745285224 | 0.034421161 | -6.365419369 | 0.0000 | 6.365419 |
| Biodiversity of Marine Artificial Structures / Management of Captive Animals | 1.933504755 | 0.200469813 | 6.359191768 | 0.0000 | 6.359192 |
| Control of Freshwater Invasive Species / Sustainable Aquaculture | 0.517918481 | 0.054706698 | -6.228815857 | 0.0000 | 6.228816 |
| Natural Pest Control / Subtidal Benthic Invertebrate Conservation | 2.295505734 | 0.313683762 | 6.080830536 | 0.0000 | 6.080831 |
| Marine and Freshwater Mammal Conservation / Natural Pest Control | 0.422747739 | 0.060409562 | -6.025158627 | 0.0000 | 6.025159 |
| Bee Conservation / Grassland Conservation | 1.770920944 | 0.168272416 | 6.014537888 | 0.0000 | 6.014538 |
| Marine Fish Conservation / Subtidal Benthic Invertebrate Conservation | 0.707415047 | 0.041102359 | -5.957396237 | 0.0000 | 5.957396 |
| Reptile Conservation / Soil Fertility | 0.584232007 | 0.052765172 | -5.950888248 | 0.0000 | 5.950888 |
| Control of Freshwater Invasive Species / Management of Captive Animals | 0.621735738 | 0.049721823 | -5.94253708 | 0.0000 | 5.942537 |
| Mediterranean Farmland / Natural Pest Control | 0.440361001 | 0.061291097 | -5.892644868 | 0.0000 | 5.892645 |
| Butterfly and Moth Conservation / Sustainable Aquaculture | 0.555869865 | 0.055472682 | -5.884310678 | 0.0000 | 5.884311 |
| Bat Conservation / Grassland Conservation | 1.677682283 | 0.150422654 | 5.770773318 | 0.0000 | 5.770773 |
| Bird Conservation / Mediterranean Farmland | 0.737284797 | 0.039233473 | -5.727518002 | 0.0000 | 5.727518 |
| Biodiversity of Marine Artificial Structures / Soil Fertility | 1.958428442 | 0.231848034 | 5.677609798 | 0.0000 | 5.67761 |
| Peatland Conservation / Subtidal Benthic Invertebrate Conservation | 0.711063593 | 0.042749167 | -5.671876632 | 0.0000 | 5.671877 |
| Butterfly and Moth Conservation / Management of Captive Animals | 0.667294514 | 0.048106901 | -5.61118034 | 0.0000 | 5.61118 |
| Marine Fish Conservation / Mediterranean Farmland | 0.699821146 | 0.044670656 | -5.591758086 | 0.0000 | 5.591758 |
| Marine Fish Conservation / Sustainable Aquaculture | 0.565645003 | 0.058191263 | -5.538599068 | 0.0000 | 5.538599 |
| Control of Freshwater Invasive Species / Marsh and Swamp Conservation | 0.746591185 | 0.040134922 | -5.436212225 | 0.0000 | 5.436212 |
| Management of Captive Animals / Natural Pest Control | 0.453844976 | 0.065955079 | -5.436084017 | 0.0000 | 5.436084 |
| Peatland Conservation / Sustainable Aquaculture | 0.568562359 | 0.059146907 | -5.427764558 | 0.0000 | 5.427765 |
| Amphibian Conservation / Grassland Conservation | 1.522881928 | 0.118128544 | 5.422322469 | 0.0000 | 5.422322 |
| Butterfly and Moth Conservation / Marsh and Swamp Conservation | 0.801298962 | 0.03295721 | -5.385913343 | 0.0000 | 5.385913 |
| Control of Freshwater Invasive Species / Marine and Freshwater Mammal Conservation | 0.667470492 | 0.050373129 | -5.356659175 | 0.0000 | 5.356659 |
| Mediterranean Farmland / Peatland Conservation | 1.421604499 | 0.093361853 | 5.356585976 | 0.0000 | 5.356586 |
| Bird Conservation / Sustainable Aquaculture | 0.595925778 | 0.057594343 | -5.355986392 | 0.0000 | 5.355986 |
| Control of Freshwater Invasive Species / Terrestrial Mammal Conservation | 0.756790208 | 0.040459 | -5.212539164 | 0.0000 | 5.212539 |
| Natural Pest Control / Soil Fertility | 2.23179824 | 0.348506334 | 5.141096486 | 0.0000 | 5.141096 |
| Forest Conservation / Sustainable Aquaculture | 0.580818443 | 0.061718941 | -5.112993939 | 0.0000 | 5.112994 |
| Butterfly and Moth Conservation / Terrestrial Mammal Conservation | 0.812245337 | 0.033092366 | -5.104159982 | 0.0000 | 5.10416 |
| Management of Captive Animals / Marine Fish Conservation | 1.472690961 | 0.112368174 | 5.073196927 | 0.000102954 | 5.073197 |
| Farmland Conservation / Grassland Conservation | 1.499976844 | 0.120374688 | 5.052267451 | 0.000114702 | 5.052267 |
| Forest Conservation / Subtidal Benthic Invertebrate Conservation | 0.726391472 | 0.046421195 | -5.002085743 | 0.000148333 | 5.002086 |
| Bee Conservation / Subtidal Benthic Invertebrate Conservation | 1.497849886 | 0.121592695 | 4.977085944 | 0.000168429 | 4.977086 |
| Butterfly and Moth Conservation / Marine and Freshwater Mammal Conservation | 0.716380561 | 0.048041625 | -4.973692123 | 0.00017135 | 4.973692 |
| Bird Conservation / Management of Captive Animals | 0.715379674 | 0.048408963 | -4.949715648 | 0.000193412 | 4.949716 |
| Biodiversity of Marine Artificial Structures / Farmland Conservation | 1.58773306 | 0.148698981 | 4.936284708 | 0.000206931 | 4.936285 |
| Management of Captive Animals / Peatland Conservation | 1.465134421 | 0.114058956 | 4.906266944 | 0.000240484 | 4.906267 |
| Amphibian Conservation / Biodiversity of Marine Artificial Structures | 0.639446464 | 0.058424302 | -4.894025187 | 0.000255608 | 4.894025 |
| Grassland Conservation / Reptile Conservation | 1.407538619 | 0.098366902 | 4.891447583 | 0.000258906 | 4.891448 |
| Shrubland and Heathland Conservation / Sustainable Aquaculture | 0.593191764 | 0.064162656 | -4.828151393 | 0.000353874 | 4.828151 |
| Forest Conservation / Mediterranean Farmland | 0.718593864 | 0.049695585 | -4.778407757 | 0.000450929 | 4.778408 |
| Bee Conservation / Marine and Freshwater Mammal Conservation | 1.543507041 | 0.140924453 | 4.754109145 | 0.00050708 | 4.754109 |
| Control of Freshwater Invasive Species / Soil Fertility | 0.629750172 | 0.061896866 | -4.704869701 | 0.000641875 | 4.70487 |
| Bat Conservation / Subtidal Benthic Invertebrate Conservation | 1.418988366 | 0.106189965 | 4.676211614 | 0.000735308 | 4.676212 |
| Primate Conservation / Sustainable Aquaculture | 0.611810472 | 0.064358041 | -4.670784061 | 0.000754396 | 4.670784 |
| Bird Conservation / Marsh and Swamp Conservation | 0.859040467 | 0.028145658 | -4.637374717 | 0.000882637 | 4.637375 |
| Bee Conservation / Mediterranean Farmland | 1.481770889 | 0.126502429 | 4.606144784 | 0.001020937 | 4.606145 |
| Primate Conservation / Reptile Conservation | 1.273322601 | 0.067008586 | 4.591539435 | 0.001092423 | 4.591539 |
| Forest Conservation / Management of Captive Animals | 0.697244058 | 0.056343462 | -4.46262951 | 0.001963255 | 4.46263 |
| Shrubland and Heathland Conservation / Subtidal Benthic Invertebrate Conservation | 0.741865971 | 0.049722457 | -4.454954864 | 0.002031686 | 4.454955 |
| Bat Conservation / Marine and Freshwater Mammal Conservation | 1.462241681 | 0.125329826 | 4.433174043 | 0.002238279 | 4.433174 |
| Marine and Freshwater Mammal Conservation / Marine Fish Conservation | 1.371782892 | 0.098174279 | 4.417002514 | 0.002404229 | 4.417003 |
| Primate Conservation / Subtidal Benthic Invertebrate Conservation | 0.765151166 | 0.047525755 | -4.309602029 | 0.003834091 | 4.309602 |
| Mediterranean Farmland / Shrubland and Heathland Conservation | 1.362579283 | 0.098169539 | 4.294142687 | 0.00409558 | 4.294143 |
| Bird Conservation / Terrestrial Mammal Conservation | 0.870775635 | 0.028105042 | -4.287132286 | 0.00421954 | 4.287132 |
| Farmland Conservation / Natural Pest Control | 0.55268196 | 0.076463281 | -4.28604727 | 0.004239034 | 4.286047 |
| Bat Conservation / Mediterranean Farmland | 1.403755925 | 0.111409416 | 4.273299996 | 0.00447441 | 4.2733 |
| Butterfly and Moth Conservation / Soil Fertility | 0.675896219 | 0.062175971 | -4.258223563 | 0.004768433 | 4.258224 |
| Marine and Freshwater Mammal Conservation / Peatland Conservation | 1.364744123 | 0.099919215 | 4.247334445 | 0.004991827 | 4.247334 |
| Marine Fish Conservation / Marsh and Swamp Conservation | 0.815390045 | 0.039242481 | -4.240605818 | 0.005134673 | 4.240606 |
| Bird Conservation / Marine and Freshwater Mammal Conservation | 0.768002856 | 0.047832729 | -4.238174214 | 0.00518722 | 4.238174 |
| Amphibian Conservation / Subtidal Benthic Invertebrate Conservation | 1.288057793 | 0.077049209 | 4.231752059 | 0.005328405 | 4.231752 |
| Amphibian Conservation / Natural Pest Control | 0.561121575 | 0.076767647 | -4.22347151 | 0.005515694 | 4.223472 |
| Mediterranean Farmland / Primate Conservation | 1.321113065 | 0.089178635 | 4.125387762 | 0.008247654 | 4.125388 |
| Management of Captive Animals / Shrubland and Heathland Conservation | 1.404301836 | 0.116972173 | 4.076328622 | 0.01003767 | 4.076329 |
| Marine Fish Conservation / Terrestrial Mammal Conservation | 0.826528915 | 0.039504935 | -3.986099489 | 0.014282006 | 3.986099 |
| Marsh and Swamp Conservation / Peatland Conservation | 1.220114098 | 0.061670594 | 3.935989996 | 0.017286883 | 3.93599 |
| Marine Fish Conservation / Soil Fertility | 0.687782057 | 0.065562612 | -3.926404234 | 0.017922739 | 3.926404 |
| Management of Captive Animals / Primate Conservation | 1.361565912 | 0.10810536 | 3.887203165 | 0.020747446 | 3.887203 |
| Amphibian Conservation / Marine and Freshwater Mammal Conservation | 1.327320108 | 0.096851222 | 3.880658879 | 0.021256013 | 3.880659 |
| Sustainable Aquaculture / Terrestrial Mammal Conservation | 1.461214914 | 0.143542079 | 3.860835705 | 0.02286517 | 3.860836 |
| Biodiversity of Marine Artificial Structures / Sustainable Aquaculture | 1.610648678 | 0.200594005 | 3.827107205 | 0.025853851 | 3.827107 |
| Peatland Conservation / Soil Fertility | 0.691329345 | 0.066760191 | -3.822586217 | 0.026279747 | 3.822586 |
| Bee Conservation / Management of Captive Animals | 1.437746689 | 0.136667433 | 3.819585048 | 0.026565893 | 3.819585 |
| Forest Conservation / Marine and Freshwater Mammal Conservation | 0.748533189 | 0.057153908 | -3.793353142 | 0.02918679 | 3.793353 |
| Natural Pest Control / Sustainable Aquaculture | 1.835473183 | 0.295278264 | 3.775039504 | 0.031149384 | 3.77504 |
| Farmland Conservation / Subtidal Benthic Invertebrate Conservation | 1.268684608 | 0.080253561 | 3.762105385 | 0.032604579 | 3.762105 |
| Marsh and Swamp Conservation / Sustainable Aquaculture | 0.69371095 | 0.068258416 | -3.716611702 | 0.038207554 | 3.716612 |
| Amphibian Conservation / Mediterranean Farmland | 1.274230855 | 0.083330985 | 3.705711693 | 0.039668532 | 3.705712 |
| Peatland Conservation / Terrestrial Mammal Conservation | 0.830791799 | 0.041730525 | -3.690557649 | 0.041780258 | 3.690558 |
| Bird Conservation / Soil Fertility | 0.724601218 | 0.064175289 | -3.637203046 | 0.0500082 | 3.637203 |
| Reptile Conservation / Shrubland and Heathland Conservation | 0.80999688 | 0.047254372 | -3.612078394 | 0.054341262 | 3.612078 |
| Butterfly and Moth Conservation / Reptile Conservation | 1.156896938 | 0.047169384 | 3.574516491 | 0.061413705 | 3.574516 |
| Farmland Conservation / Marine and Freshwater Mammal Conservation | 1.307356395 | 0.099113915 | 3.535131945 | 0.069648745 | 3.535132 |
| Forest Conservation / Soil Fertility | 0.706231826 | 0.069895475 | -3.514329279 | 0.07435875 | 3.514329 |
| Forest Conservation / Reptile Conservation | 1.208820843 | 0.066157629 | 3.465167719 | 0.086546557 | 3.465168 |
| Bat Conservation / Management of Captive Animals | 1.362049591 | 0.122176396 | 3.44469602 | 0.09208269 | 3.444696 |
| Bat Conservation / Biodiversity of Marine Artificial Structures | 0.704445949 | 0.071735202 | -3.440405456 | 0.093278711 | 3.440405 |
| Marine Fish Conservation / Reptile Conservation | 1.177241318 | 0.056298739 | 3.41206545 | 0.101498109 | 3.412065 |
| Grassland Conservation / Sustainable Aquaculture | 0.676299051 | 0.07764121 | -3.406876706 | 0.103064201 | 3.406877 |
| Marine and Freshwater Mammal Conservation / Shrubland and Heathland Conservation | 1.308079757 | 0.103314441 | 3.400281646 | 0.10508262 | 3.400282 |
| Bee Conservation / Soil Fertility | 1.456279846 | 0.161514045 | 3.389141448 | 0.108563661 | 3.389141 |
| Peatland Conservation / Reptile Conservation | 1.183313028 | 0.059467473 | 3.349277447 | 0.121775553 | 3.349277 |
| Bat Conservation / Natural Pest Control | 0.618159364 | 0.089021573 | -3.340091603 | 0.124991541 | 3.340092 |
| Control of Freshwater Invasive Species / Grassland Conservation | 0.765812816 | 0.06120637 | -3.338415009 | 0.125585594 | 3.338415 |
| Farmland Conservation / Mediterranean Farmland | 1.255065636 | 0.086043483 | 3.313855757 | 0.13454093 | 3.313856 |
| Shrubland and Heathland Conservation / Soil Fertility | 0.72127686 | 0.072856374 | -3.234643394 | 0.166769517 | 3.234643 |
| Forest Conservation / Marsh and Swamp Conservation | 0.837262901 | 0.0460452 | -3.229701621 | 0.168954438 | 3.229702 |
| Marine and Freshwater Mammal Conservation / Primate Conservation | 1.268272077 | 0.094942555 | 3.174674562 | 0.194706283 | 3.174675 |
| Subtidal Benthic Invertebrate Conservation / Terrestrial Mammal Conservation | 1.168379041 | 0.057745339 | 3.148653332 | 0.207802881 | 3.148653 |
| Bat Conservation / Soil Fertility | 1.379606981 | 0.146726356 | 3.02573916 | 0.2776645 | 3.025739 |
| Primate Conservation / Soil Fertility | 0.743915818 | 0.072770532 | -3.024173042 | 0.278638396 | 3.024173 |
| Forest Conservation / Terrestrial Mammal Conservation | 0.848700571 | 0.046428547 | -2.998765908 | 0.294721984 | 2.998766 |
| Mediterranean Farmland / Terrestrial Mammal Conservation | 1.18105736 | 0.066194748 | 2.969115881 | 0.314153637 | 2.969116 |
| Bee Conservation / Natural Pest Control | 0.652514112 | 0.096184591 | -2.89623276 | 0.364757932 | 2.896233 |
| Marsh and Swamp Conservation / Subtidal Benthic Invertebrate Conservation | 0.867578714 | 0.043156347 | -2.855633678 | 0.394526342 | 2.855634 |
| Management of Captive Animals / Terrestrial Mammal Conservation | 1.217221662 | 0.085106838 | 2.811412186 | 0.428045369 | 2.811412 |
| Bee Conservation / Biodiversity of Marine Artificial Structures | 0.743596149 | 0.079252409 | -2.779672167 | 0.452698396 | 2.779672 |
| Bird Conservation / Control of Freshwater Invasive Species | 1.150616942 | 0.058082393 | 2.779320182 | 0.452974209 | 2.77932 |
| Amphibian Conservation / Management of Captive Animals | 1.23637278 | 0.095961263 | 2.733769179 | 0.489040771 | 2.733769 |
| Butterfly and Moth Conservation / Grassland Conservation | 0.821929091 | 0.059210514 | -2.722172645 | 0.498322471 | 2.722173 |
| Marsh and Swamp Conservation / Mediterranean Farmland | 0.858265501 | 0.048345562 | -2.71335832 | 0.505398444 | 2.713358 |
| Marsh and Swamp Conservation / Shrubland and Heathland Conservation | 1.169454791 | 0.06851604 | 2.671837182 | 0.538907849 | 2.671837 |
| Management of Captive Animals / Marsh and Swamp Conservation | 1.200817549 | 0.084231894 | 2.608901941 | 0.589846468 | 2.608902 |
| Control of Freshwater Invasive Species / Primate Conservation | 0.846534187 | 0.055337499 | -2.548661743 | 0.638070783 | 2.548662 |
| Farmland Conservation / Management of Captive Animals | 1.21777697 | 0.097782195 | 2.453769755 | 0.711117211 | 2.45377 |
| Shrubland and Heathland Conservation / Terrestrial Mammal Conservation | 0.866780652 | 0.050547535 | -2.45161409 | 0.712716597 | 2.451614 |
| Marsh and Swamp Conservation / Primate Conservation | 1.133865767 | 0.05998184 | 2.374898152 | 0.76735448 | 2.374898 |
| Grassland Conservation / Marine Fish Conservation | 1.195624548 | 0.091166832 | 2.343184024 | 0.788470934 | 2.343184 |
| Amphibian Conservation / Soil Fertility | 1.252310143 | 0.120697378 | 2.334410356 | 0.794143619 | 2.33441 |
| Grassland Conservation / Management of Captive Animals | 0.811863846 | 0.074489677 | -2.271600628 | 0.832456248 | 2.271601 |
| Marine and Freshwater Mammal Conservation / Sustainable Aquaculture | 0.775942138 | 0.086708042 | -2.270134615 | 0.833300355 | 2.270135 |
| Grassland Conservation / Peatland Conservation | 1.189489666 | 0.092541078 | 2.230419634 | 0.855258206 | 2.23042 |
| Grassland Conservation / Mediterranean Farmland | 0.836723341 | 0.068340051 | -2.182553362 | 0.879334859 | 2.182553 |
| Grassland Conservation / Subtidal Benthic Invertebrate Conservation | 0.845802796 | 0.065364449 | -2.167015726 | 0.886579251 | 2.167016 |
| Subtidal Benthic Invertebrate Conservation / Sustainable Aquaculture | 0.799594249 | 0.082869038 | -2.157982675 | 0.89066164 | 2.157983 |
| Primate Conservation / Terrestrial Mammal Conservation | 0.893986586 | 0.047023114 | -2.130530232 | 0.902485905 | 2.13053 |
| Farmland Conservation / Soil Fertility | 1.233474625 | 0.121560417 | 2.129198465 | 0.903037285 | 2.129198 |
| Soil Fertility / Terrestrial Mammal Conservation | 1.201730847 | 0.108512928 | 2.035089629 | 0.936906383 | 2.03509 |
| Mediterranean Farmland / Sustainable Aquaculture | 0.808270808 | 0.08644941 | -1.990146652 | 0.949692829 | 1.990147 |
| Bee Conservation / Farmland Conservation | 1.180632189 | 0.099181671 | 1.976615538 | 0.953142247 | 1.976616 |
| Marine and Freshwater Mammal Conservation / Terrestrial Mammal Conservation | 1.133818225 | 0.073380636 | 1.940528935 | 0.961486411 | 1.940529 |
| Control of Freshwater Invasive Species / Shrubland and Heathland Conservation | 0.873104638 | 0.061162388 | -1.937141264 | 0.962208146 | 1.937141 |
| Bird Conservation / Butterfly and Moth Conservation | 1.072059877 | 0.039390138 | 1.893773003 | 0.970571191 | 1.893773 |
| Forest Conservation / Grassland Conservation | 0.858818953 | 0.069359046 | -1.884538484 | 0.972151655 | 1.884538 |
| Marsh and Swamp Conservation / Soil Fertility | 0.84350068 | 0.076313799 | -1.881170083 | 0.972711504 | 1.88117 |
| Bird Conservation / Grassland Conservation | 0.8811572 | 0.059576788 | -1.871254537 | 0.974308974 | 1.871255 |
| Amphibian Conservation / Bee Conservation | 0.859937838 | 0.070040972 | -1.852636605 | 0.977110841 | 1.852637 |
| Grassland Conservation / Soil Fertility | 0.822329112 | 0.088884812 | -1.80975315 | 0.982653324 | 1.809753 |
| Butterfly and Moth Conservation / Primate Conservation | 0.908565463 | 0.050382412 | -1.729191388 | 0.990147858 | 1.729191 |
| Marine and Freshwater Mammal Conservation / Marsh and Swamp Conservation | 1.118538115 | 0.072665406 | 1.724362793 | 0.990494867 | 1.724363 |
| Control of Freshwater Invasive Species / Forest Conservation | 0.891704606 | 0.059811216 | -1.708835072 | 0.991543196 | 1.708835 |
| Management of Captive Animals / Sustainable Aquaculture | 0.833020283 | 0.095659321 | -1.590964105 | 0.996789511 | 1.590964 |
| Grassland Conservation / Shrubland and Heathland Conservation | 1.14010189 | 0.094912726 | 1.574999168 | 0.997217046 | 1.574999 |
| Grassland Conservation / Marine and Freshwater Mammal Conservation | 0.871584385 | 0.076572223 | -1.5644422 | 0.997472103 | 1.564442 |
| Bee Conservation / Sustainable Aquaculture | 1.197672154 | 0.140678147 | 1.535674658 | 0.998067651 | 1.535675 |
| Soil Fertility / Sustainable Aquaculture | 0.822418958 | 0.105487441 | -1.524231611 | 0.998268327 | 1.524232 |
| Control of Freshwater Invasive Species / Peatland Conservation | 0.910926431 | 0.057823291 | -1.469705153 | 0.998996012 | 1.469705 |
| Bat Conservation / Farmland Conservation | 1.118472122 | 0.087123487 | 1.437363721 | 0.999286622 | 1.437364 |
| Control of Freshwater Invasive Species / Marine Fish Conservation | 0.915624602 | 0.056374667 | -1.431693258 | 0.999329094 | 1.431693 |
| Control of Freshwater Invasive Species / Reptile Conservation | 1.077911113 | 0.057649972 | 1.402781176 | 0.999512807 | 1.402781 |
| Marine Fish Conservation / Primate Conservation | 0.924542859 | 0.056234653 | -1.289877503 | 0.999876089 | 1.289878 |
| Amphibian Conservation / Bat Conservation | 0.907729636 | 0.06819671 | -1.288568439 | 0.999878186 | 1.288568 |
| Grassland Conservation / Primate Conservation | 1.105406138 | 0.087713418 | 1.262929447 | 0.999913302 | 1.262929 |
| Butterfly and Moth Conservation / Control of Freshwater Invasive Species | 1.073276752 | 0.060392301 | 1.25675322 | 0.999920256 | 1.256753 |
| Bird Conservation / Marine Fish Conservation | 1.053533179 | 0.046815336 | 1.173572134 | 0.99997583 | 1.173572 |
| Peatland Conservation / Primate Conservation | 0.929311258 | 0.058318673 | -1.16822358 | 0.999977716 | 1.168224 |
| Bat Conservation / Sustainable Aquaculture | 1.134614936 | 0.128403083 | 1.115972411 | 0.999990232 | 1.115972 |
| Butterfly and Moth Conservation / Shrubland and Heathland Conservation | 0.93708291 | 0.057070394 | -1.067014571 | 0.999995725 | 1.067015 |
| Bird Conservation / Peatland Conservation | 1.048127384 | 0.049308425 | 0.99916722 | 0.999998762 | 0.999167 |
| Biodiversity of Marine Artificial Structures / Natural Pest Control | 0.87751142 | 0.134354311 | -0.853417363 | 0.999999944 | 0.853417 |
| Management of Captive Animals / Marine and Freshwater Mammal Conservation | 1.073559795 | 0.094361885 | 0.80754338 | 0.999999982 | 0.807543 |
| Forest Conservation / Primate Conservation | 0.949343742 | 0.063028281 | -0.782997698 | 0.99999999 | 0.782998 |
| Butterfly and Moth Conservation / Forest Conservation | 0.957045823 | 0.05498586 | -0.76416276 | 0.999999994 | 0.764163 |
| Marine Fish Conservation / Shrubland and Heathland Conservation | 0.953561795 | 0.062773559 | -0.722324229 | 0.999999998 | 0.722324 |
| Peatland Conservation / Shrubland and Heathland Conservation | 0.958479861 | 0.06481077 | -0.627148764 | 1 | 0.627149 |
| Bat Conservation / Bee Conservation | 0.947350185 | 0.088138437 | -0.581344871 | 1 | 0.581345 |
| Marine and Freshwater Mammal Conservation / Soil Fertility | 0.94348766 | 0.098911919 | -0.554883159 | 1 | 0.554883 |
| Bird Conservation / Primate Conservation | 0.974036578 | 0.048280033 | -0.530724932 | 1 | 0.530725 |
| Management of Captive Animals / Subtidal Benthic Invertebrate Conservation | 1.041803746 | 0.080561361 | 0.529603698 | 1 | 0.529604 |
| Marine and Freshwater Mammal Conservation / Mediterranean Farmland | 0.960002675 | 0.074227401 | -0.527925647 | 1 | 0.527926 |
| Bird Conservation / Forest Conservation | 1.026010427 | 0.053143335 | 0.495749895 | 1 | 0.49575 |
| Primate Conservation / Shrubland and Heathland Conservation | 1.031387334 | 0.071575251 | 0.445333293 | 1 | 0.445333 |
| Butterfly and Moth Conservation / Peatland Conservation | 0.977676161 | 0.052021687 | -0.424299723 | 1 | 0.4243 |
| Forest Conservation / Marine Fish Conservation | 1.026825022 | 0.064334732 | 0.422503327 | 1 | 0.422503 |
| Marine and Freshwater Mammal Conservation / Subtidal Benthic Invertebrate Conservation | 0.970419859 | 0.07051286 | -0.413233414 | 1 | 0.413233 |
| Management of Captive Animals / Mediterranean Farmland | 1.030620276 | 0.08422326 | 0.369071007 | 1 | 0.369071 |
| Marsh and Swamp Conservation / Terrestrial Mammal Conservation | 1.013660786 | 0.037699369 | 0.364825018 | 1 | 0.364825 |
| Grassland Conservation / Marsh and Swamp Conservation | 0.974900354 | 0.068332462 | -0.362667752 | 1 | 0.362668 |
| Butterfly and Moth Conservation / Marine Fish Conservation | 0.982718598 | 0.050038504 | -0.342360568 | 1 | 0.342361 |
| Forest Conservation / Peatland Conservation | 1.02155627 | 0.065920802 | 0.330501964 | 1 | 0.330502 |
| Forest Conservation / Shrubland and Heathland Conservation | 0.979141111 | 0.069525244 | -0.296867907 | 1 | 0.296868 |
| Soil Fertility / Subtidal Benthic Invertebrate Conservation | 1.028545365 | 0.098893815 | 0.292727723 | 1 | 0.292728 |
| Amphibian Conservation / Sustainable Aquaculture | 1.029923603 | 0.10695947 | 0.283910473 | 1 | 0.28391 |
| Amphibian Conservation / Farmland Conservation | 1.015270292 | 0.064576814 | 0.23826344 | 1 | 0.238263 |
| Mediterranean Farmland / Soil Fertility | 0.982796907 | 0.098000423 | -0.174022353 | 1 | 0.174022 |
| Grassland Conservation / Terrestrial Mammal Conservation | 0.98821826 | 0.069041807 | -0.169637235 | 1 | 0.169637 |
| Mediterranean Farmland / Subtidal Benthic Invertebrate Conservation | 1.010851203 | 0.065762585 | 0.165897759 | 1 | 0.165898 |
| Farmland Conservation / Sustainable Aquaculture | 1.014432916 | 0.107398202 | 0.135352108 | 1 | 0.135352 |
| Management of Captive Animals / Soil Fertility | 1.012890419 | 0.109516884 | 0.118457953 | 1 | 0.118458 |
| Marine Fish Conservation / Peatland Conservation | 0.99486889 | 0.058484859 | -0.087508524 | 1 | 0.087509 |
| Bird Conservation / Shrubland and Heathland Conservation | 1.004608989 | 0.055852738 | 0.08271026 | 1 | 0.08271 |

Table S6 – Estimated Marginal Means comparisons of LLM accuracy between exam types for the logistic regression on the unfiltered dataset (see Table S4).

| contrast | odds.ratio | SE | z.ratio (red = negative, green = positive) | p.value (red p<0.05) | abs.z.ratio |
| --- | --- | --- | --- | --- | --- |
| closed_book / oracle | 0.136890971 | 0.004517196 | -60.26245711 | 0.0000 | 60.26246 |
| closed_book / confused | 0.175240875 | 0.005347472 | -57.07340625 | 0.0000 | 57.07341 |
| closed_book / hybrid_retrieval | 0.30906822 | 0.008113283 | -44.72983722 | 0.0000 | 44.72984 |
| oracle / sparse_retrieval | 3.550507371 | 0.121895016 | 36.90728581 | 0.0000 | 36.90729 |
| closed_book / dense_retrieval | 0.400232005 | 0.009950977 | -36.8302321 | 0.0000 | 36.83023 |
| confused / sparse_retrieval | 2.773510469 | 0.088623937 | 31.92474294 | 0.0000 | 31.92474 |
| dense_retrieval / oracle | 0.342029046 | 0.0119463 | -30.71655282 | 0.0000 | 30.71655 |
| closed_book / sparse_retrieval | 0.4860324 | 0.011670048 | -30.04808951 | 0.0000 | 30.04809 |
| confused / dense_retrieval | 2.283896413 | 0.074440385 | 25.33881395 | 0.0000 | 25.33881 |
| hybrid_retrieval / oracle | 0.442915064 | 0.015910803 | -22.67012838 | 0.0000 | 22.67013 |
| confused / hybrid_retrieval | 1.763676545 | 0.059362281 | 16.85769259 | 0.0000 | 16.85769 |
| hybrid_retrieval / sparse_retrieval | 1.5725732 | 0.043900706 | 16.21670364 | 0.0000 | 16.2167 |
| dense_retrieval / hybrid_retrieval | 0.772222652 | 0.022122614 | -9.022709973 | 0.0000 | 9.02271 |
| dense_retrieval / sparse_retrieval | 1.214376647 | 0.032326414 | 7.296493375 | 0.0000 | 7.296493 |
| confused / oracle | 0.78115891 | 0.030573413 | -6.31032056 | 0.0000 | 6.310321 |


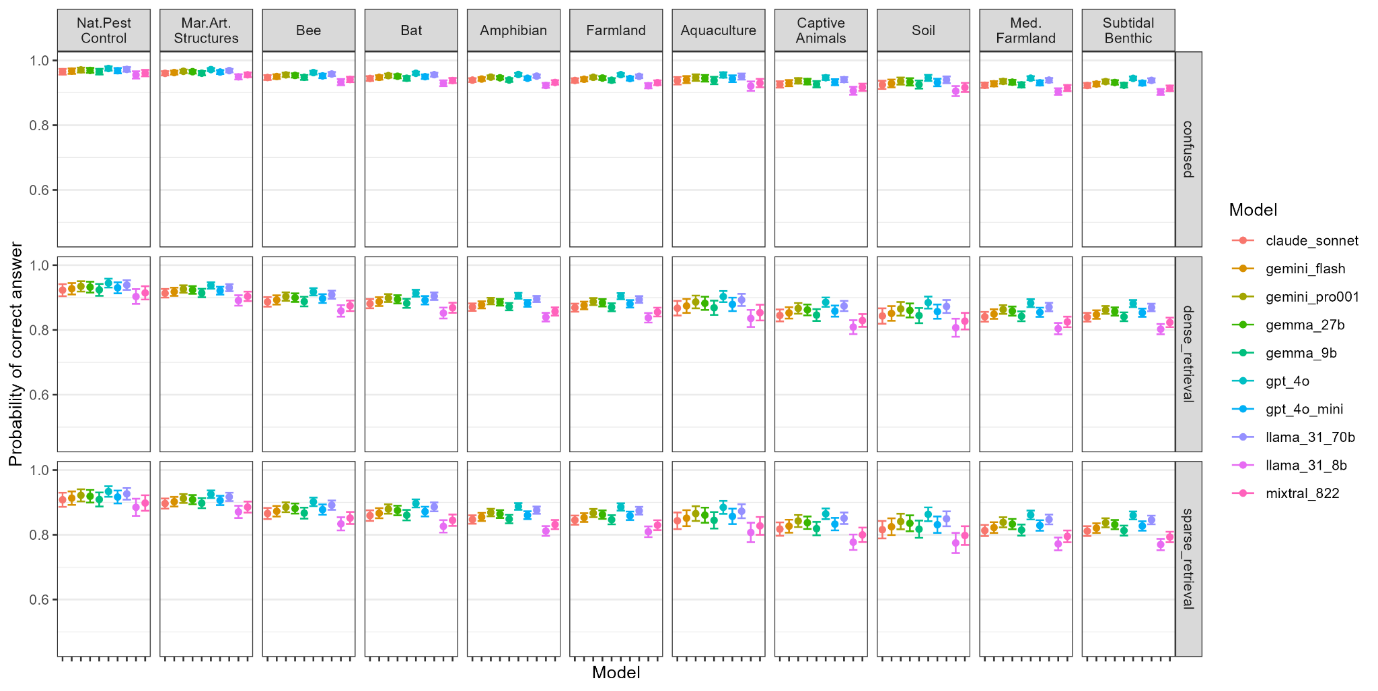

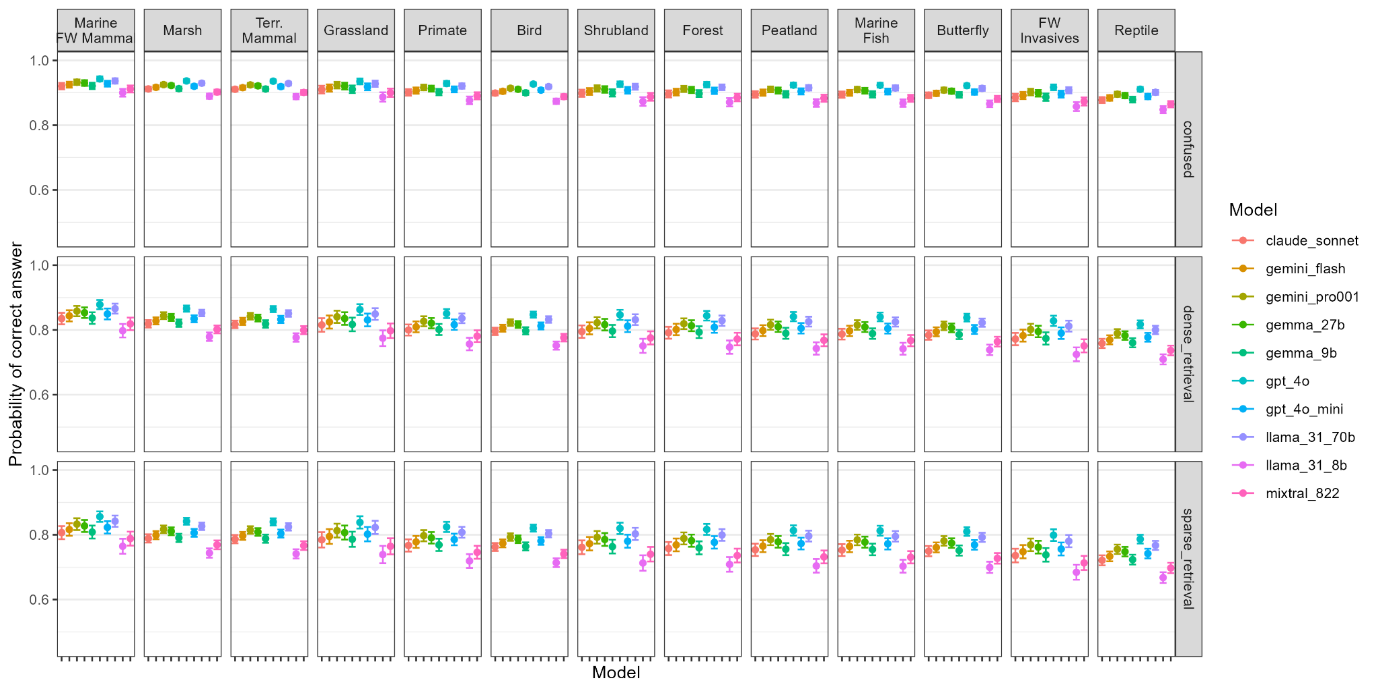
Figure S3 – Logistic regression Generalised Linear Model (GLM) predictions of the accuracy of different LLMs across different synopses, under different exam types (mean and 95% Confidence Intervals). The results for oracle, hybrid retrieval, and closed book are found in Figure 2 in the main text.

Table S7 – Estimated Marginal Means comparisons of LLM accuracy between LLMs for the unfiltered dataset (see Table S4 for logistic regression).

| contrast | odds.ratio | SE | z.ratio (red = negative, green = positive) | p.value (red p<0.05) | abs.z.ratio |
| --- | --- | --- | --- | --- | --- |
| gpt_4o / llama_31_8b | 1.831807905 | 0.06819819 | 16.25848957 | 0.0000 | 16.25849 |
| llama_31_70b / llama_31_8b | 1.639162317 | 0.059786854 | 13.54896461 | 0.0000 | 13.54896 |
| gpt_4o / mixtral_822 | 1.597978681 | 0.060359094 | 12.40965837 | 0.0000 | 12.40966 |
| gemini_pro001 / llama_31_8b | 1.535743725 | 0.055383064 | 11.89635785 | 0.0000 | 11.89636 |
| gemma_27b / llama_31_8b | 1.475281257 | 0.052845293 | 10.85548202 | 0.0000 | 10.85548 |
| gpt_4o_mini / llama_31_8b | 1.430395749 | 0.050979125 | 10.04355826 | 0.0000 | 10.04356 |
| llama_31_70b / mixtral_822 | 1.429924191 | 0.052947839 | 9.658024671 | 0.0000 | 9.658025 |
| claude_sonnet / gpt_4o | 0.701913974 | 0.026875145 | -9.244174707 | 0.0000 | 9.244175 |
| gemma_9b / gpt_4o | 0.709326071 | 0.027194516 | -8.958089676 | 0.0000 | 8.95809 |
| gemini_flash / llama_31_8b | 1.367942167 | 0.048408018 | 8.8536283 | 0.0000 | 8.853628 |
| gemini_pro001 / mixtral_822 | 1.339706923 | 0.049065484 | 7.985215571 | 0.0000 | 7.985216 |
| gemini_flash / gpt_4o | 0.746771626 | 0.028819005 | -7.566334158 | 0.0000 | 7.566334 |
| gemma_9b / llama_31_8b | 1.299349105 | 0.045618679 | 7.458612254 | 0.0000 | 7.458612 |
| claude_sonnet / llama_31_8b | 1.285771567 | 0.045070871 | 7.170711929 | 0.0000 | 7.170712 |
| gemma_27b / mixtral_822 | 1.286962454 | 0.046827451 | 6.933561421 | 0.0000 | 6.933561 |
| claude_sonnet / llama_31_70b | 0.784407715 | 0.029459051 | -6.465750081 | 0.0000 | 6.46575 |
| gpt_4o / gpt_4o_mini | 1.280630138 | 0.049715389 | 6.371603476 | 0.0000 | 6.371603 |
| gemma_9b / llama_31_70b | 0.792690932 | 0.029810728 | -6.177623323 | 0.0000 | 6.177623 |
| gpt_4o_mini / mixtral_822 | 1.247806556 | 0.045181325 | 6.114217942 | 0.0000 | 6.114218 |
| gemma_27b / gpt_4o | 0.805368976 | 0.031397763 | -5.552177118 | 0.0000 | 5.552177 |
| gemini_flash / mixtral_822 | 1.193325137 | 0.042912837 | 4.914907707 | 0.0000 | 4.914908 |
| claude_sonnet / gemini_pro001 | 0.837230552 | 0.031109577 | -4.781127658 | 0.0000 | 4.781128 |
| gemini_flash / llama_31_70b | 0.834537345 | 0.031599932 | -4.776885757 | 0.0000 | 4.776886 |
| gemini_pro001 / gpt_4o | 0.838375967 | 0.032869795 | -4.496412286 | 0.000295592 | 4.496412 |
| gemini_pro001 / gemma_9b | 1.181933107 | 0.043979172 | 4.492164692 | 0.000301445 | 4.492165 |
| llama_31_8b / mixtral_822 | 0.872350576 | 0.030081096 | -3.960347551 | 0.003000152 | 3.960348 |
| claude_sonnet / gemma_27b | 0.871543349 | 0.032180436 | -3.72363537 | 0.007516555 | 3.723635 |
| gpt_4o_mini / llama_31_70b | 0.872638259 | 0.03324708 | -3.575747218 | 0.012890972 | 3.575747 |
| gemma_9b / mixtral_822 | 1.13348794 | 0.040451048 | 3.511046998 | 0.016182845 | 3.511047 |
| gemma_27b / gemma_9b | 1.135400218 | 0.04198219 | 3.434290276 | 0.021048953 | 3.43429 |
| claude_sonnet / mixtral_822 | 1.121643567 | 0.039967471 | 3.221598946 | 0.041893334 | 3.221599 |
| gemini_flash / gemini_pro001 | 0.890735964 | 0.033376519 | -3.087937159 | 0.062576728 | 3.087937 |
| claude_sonnet / gpt_4o_mini | 0.89889219 | 0.03303335 | -2.900549648 | 0.105266444 | 2.90055 |
| gpt_4o / llama_31_70b | 1.117526853 | 0.044234501 | 2.807252985 | 0.133795002 | 2.807253 |
| gemma_27b / llama_31_70b | 0.900021457 | 0.034441438 | -2.752651263 | 0.153023524 | 2.752651 |
| gemma_9b / gpt_4o_mini | 0.908384344 | 0.033429815 | -2.610979623 | 0.212218593 | 2.61098 |
| gemini_flash / gemma_27b | 0.92724161 | 0.03452924 | -2.028566581 | 0.578614805 | 2.028567 |
| gemini_pro001 / gpt_4o_mini | 1.073649531 | 0.040484817 | 1.884593495 | 0.679687745 | 1.884593 |
| claude_sonnet / gemini_flash | 0.939931233 | 0.034312356 | -1.696980239 | 0.797587779 | 1.69698 |
| gemini_pro001 / llama_31_70b | 0.936907657 | 0.036064112 | -1.693062382 | 0.799798032 | 1.693062 |
| gemini_flash / gemma_9b | 1.052790325 | 0.038487915 | 1.407190866 | 0.925424032 | 1.407191 |
| gemini_flash / gpt_4o_mini | 0.95633825 | 0.035447391 | -1.204443852 | 0.971876041 | 1.204444 |
| gemini_pro001 / gemma_27b | 1.040983689 | 0.03942975 | 1.060424583 | 0.988294283 | 1.060425 |
| gemma_27b / gpt_4o_mini | 1.031379783 | 0.038653234 | 0.824434435 | 0.998228464 | 0.824434 |
| claude_sonnet / gemma_9b | 0.989550508 | 0.035857645 | -0.289888108 | 0.999999754 | 0.289888 |

Table S8 – Overall Large Language Model (LLM) accuracy across different exam conditions on the filtered 45-question dataset. The table is sorted by LLM performance under the hybrid retrieval strategy. Results for the unfiltered dataset are presented in Table 2.

|  | Closed Book | Oracle | Confused | Dense | Sparse | Hybrid |
| --- | --- | --- | --- | --- | --- | --- |
| GPT-4o | 75.60% | 100% | 100% | 97.80% | 91.10% | 97.80% |
| Llama 3.1 70B Instruct Turbo | 73.30% | 100% | 97.80% | 93.30% | 88.90% | 97.80% |
| Gemma2 - 27B Instruct | 71.10% | 97.80% | 100% | 91.10% | 91.10% | 95.60% |
| Claude 3.5 Sonnet | 71.10% | 97.80% | 100% | 88.90% | 82.20% | 93.30% |
| GPT-4o Mini | 71.10% | 95.60% | 95.60% | 93.30% | 93.30% | 93.30% |
| Mixtral 8x22B | 66.70% | 100% | 100% | 84.40% | 80.00% | 93.30% |
| Gemini 1.5 Pro | 73.30% | 97.80% | 97.80% | 84.40% | 86.70% | 91.10% |
| Gemini 1.5 Flash | 68.90% | 97.80% | 97.80% | 84.40% | 86.70% | 91.10% |
| Gemma2 - 9B Instruct | 68.90% | 95.60% | 95.60% | 80.00% | 82.20% | 88.90% |
| Llama 3.1 8B Instruct Turbo | 71.10% | 93.30% | 95.60% | 80.00% | 84.40% | 86.70% |

# Retrieval accuracy across unfiltered questions

Table S9 – Analysis of deviance test to test statistical significance of explanatory categorical variables Strategy and Synopsis in a logistic regression with retrieval accuracy as the response variable (see Table S10).

|  | LR Chisq | Df | Pr(>Chisq) |
| --- | --- | --- | --- |
| Strategy | 229.3425 | 2 | 0.0000 |
| Synopsis | 127.6742 | 23 | 0.0000 |

Table S10 – Model summary for logistic regression of retrieval accuracy (Retrieval Accuracy ~ Strategy + Synopsis) for the unfiltered dataset.

|  | Estimate | Std. Error | z value | Pr(>\|z\|) |
| --- | --- | --- | --- | --- |
| (Intercept) | 1.180401 | 0.148925 | 7.92615 | 0.0000 |
| StrategyHybrid | 0.508165 | 0.082311 | 6.173699 | 0.0000 |
| StrategySparse | -0.63657 | 0.07274 | -8.75134 | 0.0000 |
| Synopsis_Bat Conservation | 0.166917 | 0.251654 | 0.663281 | 0.507151 |
| Synopsis_Bee Conservation | 0.499709 | 0.287138 | 1.74031 | 0.081805 |
| Synopsis_Biodiversity of Marine Artificial Structures | 0.600311 | 0.303504 | 1.977936 | 0.047936 |
| Synopsis_Bird Conservation | -0.19911 | 0.15966 | -1.24706 | 0.212375 |
| Synopsis_Butterfly and Moth Conservation | -0.41653 | 0.18419 | -2.2614 | 0.023734 |
| Synopsis_Control of Freshwater Invasive Species | 0.19955 | 0.24574 | 0.812038 | 0.41677 |
| Synopsis_Farmland Conservation | 0.093704 | 0.21533 | 0.435162 | 0.663445 |
| Synopsis_Forest Conservation | -0.26452 | 0.231419 | -1.14303 | 0.253028 |
| Synopsis_Grassland Conservation | -0.05604 | 0.28113 | -0.19933 | 0.842004 |
| Synopsis_Management of Captive Animals | -0.10819 | 0.267262 | -0.40482 | 0.685608 |
| Synopsis_Marine and Freshwater Mammal Conservation | 0.789926 | 0.307243 | 2.571017 | 0.01014 |
| Synopsis_Marine Fish Conservation | -0.02183 | 0.213729 | -0.10215 | 0.918638 |
| Synopsis_Marsh and Swamp Conservation | 0.005618 | 0.174261 | 0.032237 | 0.974283 |
| Synopsis_Mediterranean Farmland | -0.96189 | 0.211727 | -4.54306 | 0.0000 |
| Synopsis_Natural Pest Control | -0.38626 | 0.352193 | -1.09674 | 0.272756 |
| Synopsis_Peatland Conservation | 0.199174 | 0.229222 | 0.868912 | 0.384895 |
| Synopsis_Primate Conservation | -0.16857 | 0.223557 | -0.75402 | 0.450837 |
| Synopsis_Reptile Conservation | -0.47709 | 0.173628 | -2.74776 | 0.006 |
| Synopsis_Shrubland and Heathland Conservation | -0.28202 | 0.240657 | -1.17186 | 0.241251 |
| Synopsis_Soil Fertility | -0.55142 | 0.314222 | -1.75486 | 0.079283 |
| Synopsis_Subtidal Benthic Invertebrate Conservation | 0.598126 | 0.228986 | 2.612067 | 0.009 |
| Synopsis_Sustainable Aquaculture | 0.219145 | 0.360291 | 0.608246 | 0.543024 |
| Synopsis_Terrestrial Mammal Conservation | 0.314409 | 0.177931 | 1.76703 | 0.077223 |

Table S11 – Estimated Marginal Means comparisons of retrieval accuracy between retrieval strategies for the unfiltered dataset (see Table S10 for logistic regression).

| contrast | odds.ratio | SE | z.ratio | p.value |
| --- | --- | --- | --- | --- |
| Hybrid / Dense | 1.662236 | 0.049518 | 6.1737 | 0.0000 |
| Dense / Sparse | 1.889991 | 0.137478 | 8.751341 | 0.0000 |
| Hybrid / Sparse | 3.141614 | 0.247291 | 14.54286 | 0.0000 |

Table S12 – Estimated Marginal Means comparisons for retrieval accuracy between synopses across the unfiltered dataset (see Table S10 for logistic regression).

| contrast | odds.ratio | SE | z.ratio | p.value | abs.z.ratio |
| --- | --- | --- | --- | --- | --- |
| Mediterranean Farmland / Terrestrial Mammal Conservation | 0.279069 | 0.053016 | -6.71823 | 0.0000 | 6.71823 |
| Mediterranean Farmland / Subtidal Benthic Invertebrate Conservation | 0.210134 | 0.050115 | -6.54112 | 0.0000 | 6.541118 |
| Marine and Freshwater Mammal Conservation / Mediterranean Farmland | 5.765038 | 1.812565 | 5.571806 | 0.0000 | 5.571806 |
| Reptile Conservation / Terrestrial Mammal Conservation | 0.453166 | 0.066291 | -5.41066 | 0.0000 | 5.410658 |
| Reptile Conservation / Subtidal Benthic Invertebrate Conservation | 0.341225 | 0.070081 | -5.23525 | 0.0000 | 5.235246 |
| Marsh and Swamp Conservation / Mediterranean Farmland | 2.631366 | 0.490718 | 5.188025 | 0.0000 | 5.188025 |
| Biodiversity of Marine Artificial Structures / Mediterranean Farmland | 4.769287 | 1.481946 | 5.027557 | 0.00013 | 5.027557 |
| Bee Conservation / Mediterranean Farmland | 4.312829 | 1.271226 | 4.958682 | 0.000185 | 4.958682 |
| Mediterranean Farmland / Peatland Conservation | 0.313154 | 0.074739 | -4.86479 | 0.000295 | 4.864788 |
| Butterfly and Moth Conservation / Subtidal Benthic Invertebrate Conservation | 0.362528 | 0.077718 | -4.73303 | 0.000561 | 4.733027 |
| Farmland Conservation / Mediterranean Farmland | 2.873667 | 0.647575 | 4.684267 | 0.000708 | 4.684267 |
| Butterfly and Moth Conservation / Terrestrial Mammal Conservation | 0.481458 | 0.076394 | -4.60657 | 0.001019 | 4.606568 |
| Control of Freshwater Invasive Species / Mediterranean Farmland | 3.194517 | 0.813242 | 4.562269 | 0.00125 | 4.562269 |
| Amphibian Conservation / Mediterranean Farmland | 2.616626 | 0.554009 | 4.543056 | 0.001365 | 4.543056 |
| Bird Conservation / Mediterranean Farmland | 2.144229 | 0.370699 | 4.412138 | 0.002456 | 4.412138 |
| Marine and Freshwater Mammal Conservation / Reptile Conservation | 3.550238 | 1.029868 | 4.367748 | 0.002983 | 4.367748 |
| Bat Conservation / Mediterranean Farmland | 3.091953 | 0.8048 | 4.336737 | 0.003412 | 4.336737 |
| Marine Fish Conservation / Mediterranean Farmland | 2.560118 | 0.572955 | 4.200411 | 0.00607 | 4.200411 |
| Bird Conservation / Subtidal Benthic Invertebrate Conservation | 0.450575 | 0.087272 | -4.11603 | 0.008565 | 4.11603 |
| Butterfly and Moth Conservation / Marine and Freshwater Mammal Conservation | 0.299257 | 0.088736 | -4.06869 | 0.010346 | 4.068692 |
| Bird Conservation / Terrestrial Mammal Conservation | 0.598388 | 0.077417 | -3.96917 | 0.01524 | 3.969171 |
| Biodiversity of Marine Artificial Structures / Reptile Conservation | 2.937032 | 0.840306 | 3.765719 | 0.032192 | 3.765719 |
| Bee Conservation / Reptile Conservation | 2.655935 | 0.713595 | 3.635547 | 0.050284 | 3.635547 |
| Bird Conservation / Marine and Freshwater Mammal Conservation | 0.371937 | 0.104858 | -3.50816 | 0.075806 | 3.508158 |
| Biodiversity of Marine Artificial Structures / Butterfly and Moth Conservation | 2.764441 | 0.808969 | 3.474779 | 0.084043 | 3.474779 |
| Soil Fertility / Subtidal Benthic Invertebrate Conservation | 0.316781 | 0.105433 | -3.45388 | 0.089564 | 3.453881 |
| Marine and Freshwater Mammal Conservation / Soil Fertility | 3.824176 | 1.494575 | 3.432102 | 0.095629 | 3.432102 |
| Marsh and Swamp Conservation / Reptile Conservation | 1.620453 | 0.229721 | 3.405008 | 0.103633 | 3.405008 |
| Mediterranean Farmland / Primate Conservation | 0.452341 | 0.105476 | -3.4022 | 0.104492 | 3.4022 |
| Forest Conservation / Subtidal Benthic Invertebrate Conservation | 0.422045 | 0.108082 | -3.3685 | 0.115256 | 3.3685 |
| Shrubland and Heathland Conservation / Subtidal Benthic Invertebrate Conservation | 0.414723 | 0.109682 | -3.32795 | 0.129344 | 3.327948 |
| Bee Conservation / Butterfly and Moth Conservation | 2.499862 | 0.689011 | 3.324274 | 0.130684 | 3.324274 |
| Peatland Conservation / Reptile Conservation | 1.966514 | 0.404341 | 3.289002 | 0.144095 | 3.289002 |
| Mediterranean Farmland / Sustainable Aquaculture | 0.306962 | 0.112456 | -3.22377 | 0.171607 | 3.223765 |
| Forest Conservation / Marine and Freshwater Mammal Conservation | 0.348386 | 0.114252 | -3.21529 | 0.175445 | 3.215291 |
| Marine and Freshwater Mammal Conservation / Shrubland and Heathland Conservation | 2.921051 | 0.977182 | 3.204318 | 0.180508 | 3.204318 |
| Grassland Conservation / Mediterranean Farmland | 2.474028 | 0.714617 | 3.136078 | 0.214345 | 3.136078 |
| Management of Captive Animals / Mediterranean Farmland | 2.348301 | 0.646652 | 3.100161 | 0.233795 | 3.100161 |
| Primate Conservation / Subtidal Benthic Invertebrate Conservation | 0.464547 | 0.115674 | -3.07905 | 0.245754 | 3.07905 |
| Control of Freshwater Invasive Species / Reptile Conservation | 1.967254 | 0.440431 | 3.022314 | 0.279797 | 3.022314 |
| Farmland Conservation / Reptile Conservation | 1.769668 | 0.336232 | 3.004212 | 0.29123 | 3.004212 |
| Marine and Freshwater Mammal Conservation / Primate Conservation | 2.607762 | 0.840848 | 2.972619 | 0.311821 | 2.972619 |
| Biodiversity of Marine Artificial Structures / Soil Fertility | 3.163656 | 1.22711 | 2.969311 | 0.314023 | 2.969311 |
| Forest Conservation / Mediterranean Farmland | 2.008458 | 0.483455 | 2.897133 | 0.36411 | 2.897133 |
| Soil Fertility / Terrestrial Mammal Conservation | 0.420704 | 0.126204 | -2.88624 | 0.371986 | 2.886242 |
| Marsh and Swamp Conservation / Subtidal Benthic Invertebrate Conservation | 0.552938 | 0.113838 | -2.87797 | 0.378023 | 2.877966 |
| Biodiversity of Marine Artificial Structures / Bird Conservation | 2.224244 | 0.617981 | 2.877268 | 0.378534 | 2.877268 |
| Butterfly and Moth Conservation / Peatland Conservation | 0.540262 | 0.115943 | -2.86899 | 0.384618 | 2.868992 |
| Bee Conservation / Soil Fertility | 2.860869 | 1.073415 | 2.801462 | 0.435725 | 2.801462 |
| Bat Conservation / Reptile Conservation | 1.904093 | 0.438622 | 2.795681 | 0.440208 | 2.795681 |
| Marine and Freshwater Mammal Conservation / Natural Pest Control | 3.241996 | 1.367954 | 2.787521 | 0.446561 | 2.787521 |
| Butterfly and Moth Conservation / Mediterranean Farmland | 1.725227 | 0.337647 | 2.786539 | 0.447328 | 2.786539 |
| Amphibian Conservation / Reptile Conservation | 1.611376 | 0.27978 | 2.747762 | 0.47789 | 2.747762 |
| Forest Conservation / Terrestrial Mammal Conservation | 0.560499 | 0.11864 | -2.73506 | 0.488012 | 2.735057 |
| Butterfly and Moth Conservation / Marsh and Swamp Conservation | 0.655639 | 0.101309 | -2.73199 | 0.490464 | 2.731988 |
| Mediterranean Farmland / Shrubland and Heathland Conservation | 0.506684 | 0.126468 | -2.72384 | 0.496986 | 2.72384 |
| Marine and Freshwater Mammal Conservation / Marsh and Swamp Conservation | 2.190892 | 0.636299 | 2.700514 | 0.515738 | 2.700514 |
| Shrubland and Heathland Conservation / Terrestrial Mammal Conservation | 0.550776 | 0.122125 | -2.68985 | 0.524342 | 2.68985 |
| Bee Conservation / Bird Conservation | 2.011366 | 0.522673 | 2.689198 | 0.524869 | 2.689198 |
| Natural Pest Control / Subtidal Benthic Invertebrate Conservation | 0.373667 | 0.137839 | -2.66859 | 0.541539 | 2.668587 |
| Biodiversity of Marine Artificial Structures / Forest Conservation | 2.374601 | 0.770413 | 2.665616 | 0.543944 | 2.665616 |
| Biodiversity of Marine Artificial Structures / Shrubland and Heathland Conservation | 2.416521 | 0.800091 | 2.664904 | 0.544521 | 2.664904 |
| Butterfly and Moth Conservation / Control of Freshwater Invasive Species | 0.540059 | 0.125384 | -2.6536 | 0.553676 | 2.653602 |
| Amphibian Conservation / Subtidal Benthic Invertebrate Conservation | 0.549841 | 0.125906 | -2.61207 | 0.587291 | 2.612067 |
| Mediterranean Farmland / Reptile Conservation | 0.615822 | 0.114413 | -2.6094 | 0.589442 | 2.609404 |
| Marine Fish Conservation / Subtidal Benthic Invertebrate Conservation | 0.537967 | 0.129224 | -2.58091 | 0.612366 | 2.580912 |
| Marine and Freshwater Mammal Conservation / Marine Fish Conservation | 2.251865 | 0.710907 | 2.571323 | 0.620041 | 2.571323 |
| Amphibian Conservation / Marine and Freshwater Mammal Conservation | 0.453878 | 0.139451 | -2.57102 | 0.620285 | 2.571017 |
| Butterfly and Moth Conservation / Farmland Conservation | 0.600357 | 0.119888 | -2.55505 | 0.633001 | 2.555055 |
| Management of Captive Animals / Marine and Freshwater Mammal Conservation | 0.407335 | 0.144254 | -2.53605 | 0.648031 | 2.536046 |
| Bee Conservation / Shrubland and Heathland Conservation | 2.185241 | 0.690875 | 2.472603 | 0.697014 | 2.472603 |
| Bee Conservation / Forest Conservation | 2.147333 | 0.663914 | 2.47178 | 0.697635 | 2.47178 |
| Bat Conservation / Butterfly and Moth Conservation | 1.792201 | 0.427293 | 2.447151 | 0.716018 | 2.447151 |
| Management of Captive Animals / Subtidal Benthic Invertebrate Conservation | 0.493457 | 0.14255 | -2.44503 | 0.717583 | 2.445029 |
| Marine Fish Conservation / Reptile Conservation | 1.576577 | 0.296665 | 2.419381 | 0.736238 | 2.419381 |
| Biodiversity of Marine Artificial Structures / Primate Conservation | 2.157343 | 0.687921 | 2.411224 | 0.742066 | 2.411224 |
| Primate Conservation / Terrestrial Mammal Conservation | 0.616945 | 0.125264 | -2.37874 | 0.764737 | 2.378736 |
| Biodiversity of Marine Artificial Structures / Natural Pest Control | 2.682031 | 1.124372 | 2.353333 | 0.781816 | 2.353333 |
| Grassland Conservation / Marine and Freshwater Mammal Conservation | 0.429143 | 0.156518 | -2.31948 | 0.803624 | 2.319476 |
| Amphibian Conservation / Butterfly and Moth Conservation | 1.516685 | 0.279358 | 2.261401 | 0.83828 | 2.261401 |
| Peatland Conservation / Soil Fertility | 2.118251 | 0.705306 | 2.254257 | 0.842291 | 2.254257 |
| Bird Conservation / Reptile Conservation | 1.320463 | 0.162876 | 2.253653 | 0.842627 | 2.253653 |
| Bee Conservation / Primate Conservation | 1.950869 | 0.591771 | 2.203076 | 0.869336 | 2.203076 |
| Farmland Conservation / Marine and Freshwater Mammal Conservation | 0.498465 | 0.157902 | -2.19784 | 0.871935 | 2.197836 |
| Control of Freshwater Invasive Species / Soil Fertility | 2.119049 | 0.730111 | 2.179581 | 0.880743 | 2.179581 |
| Bee Conservation / Natural Pest Control | 2.42534 | 0.988398 | 2.174005 | 0.883355 | 2.174005 |
| Grassland Conservation / Subtidal Benthic Invertebrate Conservation | 0.519877 | 0.156876 | -2.16785 | 0.886197 | 2.16785 |
| Marsh and Swamp Conservation / Terrestrial Mammal Conservation | 0.734334 | 0.107951 | -2.10056 | 0.914402 | 2.100557 |
| Farmland Conservation / Subtidal Benthic Invertebrate Conservation | 0.603854 | 0.145908 | -2.0876 | 0.919235 | 2.0876 |
| Biodiversity of Marine Artificial Structures / Marsh and Swamp Conservation | 1.812476 | 0.519221 | 2.075931 | 0.923425 | 2.075931 |
| Natural Pest Control / Terrestrial Mammal Conservation | 0.496251 | 0.1685 | -2.06356 | 0.927703 | 2.063556 |
| Bat Conservation / Soil Fertility | 2.051014 | 0.715373 | 2.059505 | 0.929067 | 2.059505 |
| Bird Conservation / Peatland Conservation | 0.671474 | 0.130237 | -2.05344 | 0.931074 | 2.053441 |
| Biodiversity of Marine Artificial Structures / Management of Captive Animals | 2.030953 | 0.71266 | 2.01911 | 0.941692 | 2.01911 |
| Reptile Conservation / Sustainable Aquaculture | 0.498459 | 0.172338 | -2.01375 | 0.943239 | 2.013745 |
| Farmland Conservation / Soil Fertility | 1.906217 | 0.616767 | 1.993848 | 0.948718 | 1.993848 |
| Butterfly and Moth Conservation / Marine Fish Conservation | 0.673886 | 0.133402 | -1.99382 | 0.948725 | 1.993821 |
| Biodiversity of Marine Artificial Structures / Marine Fish Conservation | 1.862917 | 0.581337 | 1.993683 | 0.948762 | 1.993683 |
| Amphibian Conservation / Biodiversity of Marine Artificial Structures | 0.548641 | 0.166515 | -1.97794 | 0.952814 | 1.977936 |
| Marsh and Swamp Conservation / Soil Fertility | 1.745489 | 0.519809 | 1.870489 | 0.974429 | 1.870489 |
| Bird Conservation / Control of Freshwater Invasive Species | 0.671221 | 0.143121 | -1.86965 | 0.974561 | 1.869649 |
| Bee Conservation / Marsh and Swamp Conservation | 1.639008 | 0.441008 | 1.836289 | 0.979368 | 1.836289 |
| Bat Conservation / Marine and Freshwater Mammal Conservation | 0.536328 | 0.183693 | -1.81899 | 0.98156 | 1.818994 |
| Peatland Conservation / Shrubland and Heathland Conservation | 1.618001 | 0.428219 | 1.818154 | 0.981662 | 1.818154 |
| Biodiversity of Marine Artificial Structures / Grassland Conservation | 1.927741 | 0.697021 | 1.815255 | 0.982009 | 1.815255 |
| Butterfly and Moth Conservation / Sustainable Aquaculture | 0.529579 | 0.185969 | -1.81019 | 0.982603 | 1.810186 |
| Marine and Freshwater Mammal Conservation / Peatland Conservation | 1.805346 | 0.58922 | 1.810039 | 0.98262 | 1.810039 |
| Forest Conservation / Peatland Conservation | 0.628957 | 0.161194 | -1.80926 | 0.98271 | 1.809262 |
| Bee Conservation / Management of Captive Animals | 1.836574 | 0.618638 | 1.804704 | 0.983228 | 1.804704 |
| Soil Fertility / Sustainable Aquaculture | 0.462753 | 0.200711 | -1.77658 | 0.986157 | 1.776584 |
| Amphibian Conservation / Terrestrial Mammal Conservation | 0.73022 | 0.129929 | -1.76703 | 0.987052 | 1.76703 |
| Bee Conservation / Marine Fish Conservation | 1.684622 | 0.498924 | 1.760988 | 0.987594 | 1.760988 |
| Amphibian Conservation / Soil Fertility | 1.735711 | 0.545399 | 1.754862 | 0.988124 | 1.754862 |
| Marine Fish Conservation / Terrestrial Mammal Conservation | 0.71445 | 0.137287 | -1.74982 | 0.988546 | 1.749822 |
| Control of Freshwater Invasive Species / Marine and Freshwater Mammal Conservation | 0.554119 | 0.187392 | -1.74574 | 0.988878 | 1.745744 |
| Amphibian Conservation / Bee Conservation | 0.606707 | 0.174209 | -1.74031 | 0.989309 | 1.74031 |
| Control of Freshwater Invasive Species / Shrubland and Heathland Conservation | 1.61861 | 0.451735 | 1.725503 | 0.990414 | 1.725503 |
| Control of Freshwater Invasive Species / Forest Conservation | 1.590532 | 0.431293 | 1.711402 | 0.991377 | 1.711402 |
| Management of Captive Animals / Terrestrial Mammal Conservation | 0.655339 | 0.164064 | -1.68805 | 0.992795 | 1.688054 |
| Bat Conservation / Bird Conservation | 1.441989 | 0.317259 | 1.663627 | 0.994065 | 1.663627 |
| Bird Conservation / Farmland Conservation | 0.746164 | 0.132311 | -1.65129 | 0.994631 | 1.651293 |
| Bird Conservation / Marsh and Swamp Conservation | 0.814873 | 0.101261 | -1.64747 | 0.994797 | 1.647468 |
| Marine Fish Conservation / Soil Fertility | 1.698227 | 0.547652 | 1.6422 | 0.995019 | 1.6422 |
| Marine and Freshwater Mammal Conservation / Terrestrial Mammal Conservation | 1.608845 | 0.470795 | 1.624981 | 0.995688 | 1.624981 |
| Biodiversity of Marine Artificial Structures / Farmland Conservation | 1.659652 | 0.519723 | 1.617769 | 0.995945 | 1.617769 |
| Mediterranean Farmland / Natural Pest Control | 0.562355 | 0.201518 | -1.60633 | 0.996326 | 1.60633 |
| Grassland Conservation / Reptile Conservation | 1.523561 | 0.399506 | 1.605724 | 0.996346 | 1.605724 |
| Bee Conservation / Grassland Conservation | 1.743242 | 0.60656 | 1.597205 | 0.996608 | 1.597205 |
| Natural Pest Control / Peatland Conservation | 0.556862 | 0.205488 | -1.5865 | 0.996914 | 1.586502 |
| Bat Conservation / Shrubland and Heathland Conservation | 1.566643 | 0.445412 | 1.579033 | 0.997114 | 1.579033 |
| Bird Conservation / Butterfly and Moth Conservation | 1.242868 | 0.171295 | 1.577548 | 0.997152 | 1.577548 |
| Bat Conservation / Subtidal Benthic Invertebrate Conservation | 0.649723 | 0.178343 | -1.57094 | 0.997318 | 1.570944 |
| Peatland Conservation / Subtidal Benthic Invertebrate Conservation | 0.671023 | 0.170495 | -1.57017 | 0.997336 | 1.570165 |
| Bat Conservation / Forest Conservation | 1.539466 | 0.425715 | 1.560153 | 0.99757 | 1.560153 |
| Primate Conservation / Reptile Conservation | 1.361412 | 0.271259 | 1.548431 | 0.997821 | 1.548431 |
| Control of Freshwater Invasive Species / Natural Pest Control | 1.796452 | 0.681741 | 1.543673 | 0.997916 | 1.543673 |
| Management of Captive Animals / Reptile Conservation | 1.446135 | 0.357619 | 1.491734 | 0.998743 | 1.491734 |
| Farmland Conservation / Shrubland and Heathland Conservation | 1.456041 | 0.367971 | 1.486707 | 0.998805 | 1.486707 |
| Control of Freshwater Invasive Species / Subtidal Benthic Invertebrate Conservation | 0.671275 | 0.180626 | -1.48126 | 0.998869 | 1.481263 |
| Peatland Conservation / Primate Conservation | 1.444467 | 0.359975 | 1.475624 | 0.998933 | 1.475624 |
| Farmland Conservation / Forest Conservation | 1.430783 | 0.349025 | 1.468485 | 0.999009 | 1.468485 |
| Bat Conservation / Natural Pest Control | 1.738774 | 0.666559 | 1.443017 | 0.999242 | 1.443017 |
| Grassland Conservation / Terrestrial Mammal Conservation | 0.690425 | 0.183035 | -1.39736 | 0.999542 | 1.397363 |
| Control of Freshwater Invasive Species / Primate Conservation | 1.44501 | 0.382181 | 1.391835 | 0.99957 | 1.391835 |
| Bee Conservation / Farmland Conservation | 1.50081 | 0.446218 | 1.365559 | 0.999683 | 1.365559 |
| Subtidal Benthic Invertebrate Conservation / Terrestrial Mammal Conservation | 1.328057 | 0.27753 | 1.357663 | 0.999711 | 1.357663 |
| Butterfly and Moth Conservation / Grassland Conservation | 0.697335 | 0.187813 | -1.33847 | 0.999771 | 1.338466 |
| Grassland Conservation / Soil Fertility | 1.64112 | 0.608202 | 1.336688 | 0.999776 | 1.336688 |
| Marine and Freshwater Mammal Conservation / Sustainable Aquaculture | 1.769648 | 0.758668 | 1.331388 | 0.99979 | 1.331388 |
| Farmland Conservation / Natural Pest Control | 1.61602 | 0.582646 | 1.33123 | 0.999791 | 1.33123 |
| Marsh and Swamp Conservation / Shrubland and Heathland Conservation | 1.333271 | 0.291697 | 1.314703 | 0.99983 | 1.314703 |
| Natural Pest Control / Sustainable Aquaculture | 0.545851 | 0.252174 | -1.31045 | 0.999839 | 1.310455 |
| Shrubland and Heathland Conservation / Sustainable Aquaculture | 0.605826 | 0.232519 | -1.30578 | 0.999848 | 1.305775 |
| Forest Conservation / Marsh and Swamp Conservation | 0.763276 | 0.159203 | -1.29512 | 0.999867 | 1.295123 |
| Forest Conservation / Sustainable Aquaculture | 0.616521 | 0.233095 | -1.27926 | 0.999892 | 1.279258 |
| Mediterranean Farmland / Soil Fertility | 0.663339 | 0.212989 | -1.27838 | 0.999893 | 1.278377 |
| Bat Conservation / Biodiversity of Marine Artificial Structures | 0.648305 | 0.219875 | -1.27787 | 0.999894 | 1.277867 |
| Amphibian Conservation / Bird Conservation | 1.220311 | 0.194835 | 1.24706 | 0.99993 | 1.24706 |
| Bat Conservation / Primate Conservation | 1.398617 | 0.377608 | 1.242591 | 0.999934 | 1.242591 |
| Biodiversity of Marine Artificial Structures / Peatland Conservation | 1.493522 | 0.482198 | 1.24245 | 0.999934 | 1.24245 |
| Bird Conservation / Sustainable Aquaculture | 0.658197 | 0.223092 | -1.23398 | 0.999942 | 1.23398 |
| Management of Captive Animals / Soil Fertility | 1.55772 | 0.561085 | 1.230506 | 0.999945 | 1.230506 |
| Bird Conservation / Soil Fertility | 1.422351 | 0.41175 | 1.217025 | 0.999954 | 1.217025 |
| Butterfly and Moth Conservation / Management of Captive Animals | 0.73467 | 0.187209 | -1.21 | 0.999959 | 1.21 |
| Biodiversity of Marine Artificial Structures / Control of Freshwater Invasive Species | 1.49296 | 0.499827 | 1.197055 | 0.999966 | 1.197055 |
| Butterfly and Moth Conservation / Primate Conservation | 0.78039 | 0.162727 | -1.18915 | 0.999969 | 1.18915 |
| Amphibian Conservation / Shrubland and Heathland Conservation | 1.325802 | 0.319064 | 1.171865 | 0.999976 | 1.171865 |
| Primate Conservation / Soil Fertility | 1.466459 | 0.482567 | 1.163434 | 0.999979 | 1.163434 |
| Marsh and Swamp Conservation / Natural Pest Control | 1.479761 | 0.499603 | 1.160702 | 0.99998 | 1.160702 |
| Amphibian Conservation / Forest Conservation | 1.302803 | 0.301494 | 1.143025 | 0.999985 | 1.143025 |
| Farmland Conservation / Terrestrial Mammal Conservation | 0.801952 | 0.155524 | -1.13806 | 0.999986 | 1.138058 |
| Farmland Conservation / Primate Conservation | 1.299877 | 0.307412 | 1.108995 | 0.999991 | 1.108995 |
| Amphibian Conservation / Natural Pest Control | 1.471472 | 0.518242 | 1.096738 | 0.999993 | 1.096738 |
| Management of Captive Animals / Peatland Conservation | 0.73538 | 0.21257 | -1.06333 | 0.999996 | 1.063331 |
| Primate Conservation / Sustainable Aquaculture | 0.678608 | 0.253339 | -1.03855 | 0.999997 | 1.038546 |
| Marine Fish Conservation / Shrubland and Heathland Conservation | 1.29717 | 0.326047 | 1.035141 | 0.999998 | 1.035141 |
| Bat Conservation / Bee Conservation | 0.71692 | 0.232706 | -1.02526 | 0.999998 | 1.025263 |
| Forest Conservation / Reptile Conservation | 1.236853 | 0.257293 | 1.021865 | 0.999998 | 1.021865 |
| Control of Freshwater Invasive Species / Management of Captive Animals | 1.360353 | 0.411272 | 1.017916 | 0.999998 | 1.017916 |
| Marine Fish Conservation / Natural Pest Control | 1.439694 | 0.517692 | 1.013477 | 0.999998 | 1.013477 |
| Bird Conservation / Marine Fish Conservation | 0.837551 | 0.146881 | -1.01086 | 0.999998 | 1.010856 |
| Subtidal Benthic Invertebrate Conservation / Sustainable Aquaculture | 1.460795 | 0.550139 | 1.006316 | 0.999999 | 1.006316 |
| Forest Conservation / Marine Fish Conservation | 0.784518 | 0.190263 | -1.00067 | 0.999999 | 1.000672 |
| Biodiversity of Marine Artificial Structures / Terrestrial Mammal Conservation | 1.330962 | 0.384258 | 0.990283 | 0.999999 | 0.990283 |
| Bee Conservation / Peatland Conservation | 1.350581 | 0.415339 | 0.977265 | 0.999999 | 0.977265 |
| Marsh and Swamp Conservation / Peatland Conservation | 0.824023 | 0.169864 | -0.93896 | 1 | 0.938957 |
| Bee Conservation / Control of Freshwater Invasive Species | 1.350072 | 0.43206 | 0.937914 | 1 | 0.937914 |
| Marine Fish Conservation / Peatland Conservation | 0.801712 | 0.192756 | -0.91921 | 1 | 0.919209 |
| Bat Conservation / Management of Captive Animals | 1.316677 | 0.404423 | 0.895677 | 1 | 0.895677 |
| Biodiversity of Marine Artificial Structures / Sustainable Aquaculture | 1.46399 | 0.623714 | 0.894678 | 1 | 0.894678 |
| Reptile Conservation / Shrubland and Heathland Conservation | 0.822776 | 0.179571 | -0.8938 | 1 | 0.893797 |
| Marsh and Swamp Conservation / Primate Conservation | 1.190274 | 0.237841 | 0.871702 | 1 | 0.871702 |
| Amphibian Conservation / Peatland Conservation | 0.819407 | 0.187826 | -0.86891 | 1 | 0.868912 |
| Control of Freshwater Invasive Species / Marsh and Swamp Conservation | 1.214015 | 0.272381 | 0.864369 | 1 | 0.864369 |
| Control of Freshwater Invasive Species / Marine Fish Conservation | 1.247801 | 0.319721 | 0.864008 | 1 | 0.864008 |
| Forest Conservation / Soil Fertility | 1.332289 | 0.445589 | 0.857814 | 1 | 0.857814 |
| Grassland Conservation / Peatland Conservation | 0.774752 | 0.233921 | -0.84527 | 1 | 0.845268 |
| Grassland Conservation / Natural Pest Control | 1.391281 | 0.561105 | 0.818806 | 1 | 0.818806 |
| Management of Captive Animals / Sustainable Aquaculture | 0.720839 | 0.289074 | -0.81626 | 1 | 0.816257 |
| Control of Freshwater Invasive Species / Grassland Conservation | 1.291221 | 0.406288 | 0.812283 | 1 | 0.812283 |
| Amphibian Conservation / Control of Freshwater Invasive Species | 0.819099 | 0.201285 | -0.81204 | 1 | 0.812038 |
| Shrubland and Heathland Conservation / Soil Fertility | 1.309178 | 0.44631 | 0.79024 | 1 | 0.79024 |
| Bee Conservation / Marine and Freshwater Mammal Conservation | 0.748101 | 0.27631 | -0.78575 | 1 | 0.785754 |
| Amphibian Conservation / Primate Conservation | 1.183607 | 0.264603 | 0.75402 | 1 | 0.75402 |
| Grassland Conservation / Shrubland and Heathland Conservation | 1.25355 | 0.389468 | 0.727343 | 1 | 0.727343 |
| Farmland Conservation / Management of Captive Animals | 1.223722 | 0.340406 | 0.725798 | 1 | 0.725798 |
| Bat Conservation / Marine Fish Conservation | 1.207739 | 0.316313 | 0.72068 | 1 | 0.72068 |
| Management of Captive Animals / Natural Pest Control | 1.320578 | 0.51999 | 0.70619 | 1 | 0.70619 |
| Butterfly and Moth Conservation / Forest Conservation | 0.858981 | 0.18633 | -0.70076 | 1 | 0.70076 |
| Bat Conservation / Marsh and Swamp Conservation | 1.175037 | 0.271229 | 0.698794 | 1 | 0.698794 |
| Bat Conservation / Grassland Conservation | 1.249765 | 0.399043 | 0.698274 | 1 | 0.698274 |
| Forest Conservation / Grassland Conservation | 0.811817 | 0.246462 | -0.68671 | 1 | 0.686709 |
| Bee Conservation / Terrestrial Mammal Conservation | 1.203578 | 0.326714 | 0.682621 | 1 | 0.682621 |
| Bee Conservation / Sustainable Aquaculture | 1.323875 | 0.548795 | 0.676811 | 1 | 0.676811 |
| Grassland Conservation / Sustainable Aquaculture | 0.759433 | 0.311669 | -0.67053 | 1 | 0.67053 |
| Amphibian Conservation / Bat Conservation | 0.846269 | 0.212967 | -0.66328 | 1 | 0.663281 |
| Marine Fish Conservation / Sustainable Aquaculture | 0.785859 | 0.288821 | -0.65568 | 1 | 0.655682 |
| Bat Conservation / Terrestrial Mammal Conservation | 0.862869 | 0.20157 | -0.63137 | 1 | 0.631374 |
| Marine Fish Conservation / Primate Conservation | 1.158046 | 0.272179 | 0.624311 | 1 | 0.624311 |
| Marsh and Swamp Conservation / Sustainable Aquaculture | 0.80773 | 0.279526 | -0.61702 | 1 | 0.617019 |
| Amphibian Conservation / Sustainable Aquaculture | 0.803205 | 0.289387 | -0.60825 | 1 | 0.608246 |
| Natural Pest Control / Primate Conservation | 0.804369 | 0.294 | -0.59561 | 1 | 0.595608 |
| Butterfly and Moth Conservation / Shrubland and Heathland Conservation | 0.874144 | 0.19821 | -0.59321 | 1 | 0.593214 |
| Marine and Freshwater Mammal Conservation / Subtidal Benthic Invertebrate Conservation | 1.211428 | 0.395156 | 0.588001 | 1 | 0.588001 |
| Management of Captive Animals / Shrubland and Heathland Conservation | 1.189846 | 0.354815 | 0.582906 | 1 | 0.582906 |
| Bird Conservation / Natural Pest Control | 1.205817 | 0.398299 | 0.566603 | 1 | 0.566603 |
| Bird Conservation / Grassland Conservation | 0.866695 | 0.219442 | -0.56505 | 1 | 0.565052 |
| Peatland Conservation / Terrestrial Mammal Conservation | 0.891156 | 0.186472 | -0.55071 | 1 | 0.550714 |
| Forest Conservation / Management of Captive Animals | 0.855282 | 0.248715 | -0.53757 | 1 | 0.537569 |
| Farmland Conservation / Grassland Conservation | 1.161534 | 0.338613 | 0.513653 | 1 | 0.513653 |
| Farmland Conservation / Marine Fish Conservation | 1.122475 | 0.255054 | 0.508465 | 1 | 0.508465 |
| Control of Freshwater Invasive Species / Terrestrial Mammal Conservation | 0.891492 | 0.202566 | -0.50549 | 1 | 0.505494 |
| Biodiversity of Marine Artificial Structures / Marine and Freshwater Mammal Conservation | 0.827278 | 0.316192 | -0.4961 | 1 | 0.496104 |
| Farmland Conservation / Marsh and Swamp Conservation | 1.092082 | 0.20812 | 0.462218 | 1 | 0.462218 |
| Management of Captive Animals / Marsh and Swamp Conservation | 0.892426 | 0.221097 | -0.45938 | 1 | 0.459382 |
| Butterfly and Moth Conservation / Soil Fertility | 1.144411 | 0.347541 | 0.444177 | 1 | 0.444177 |
| Primate Conservation / Shrubland and Heathland Conservation | 1.120137 | 0.290959 | 0.436766 | 1 | 0.436766 |
| Farmland Conservation / Peatland Conservation | 0.899901 | 0.217645 | -0.43609 | 1 | 0.436092 |
| Amphibian Conservation / Farmland Conservation | 0.910553 | 0.196069 | -0.43516 | 1 | 0.435162 |
| Control of Freshwater Invasive Species / Farmland Conservation | 1.111652 | 0.28632 | 0.410956 | 1 | 0.410956 |
| Amphibian Conservation / Management of Captive Animals | 1.114263 | 0.2978 | 0.404823 | 1 | 0.404823 |
| Bird Conservation / Shrubland and Heathland Conservation | 1.086446 | 0.225254 | 0.3999 | 1 | 0.3999 |
| Butterfly and Moth Conservation / Reptile Conservation | 1.062433 | 0.163351 | 0.393889 | 1 | 0.393889 |
| Natural Pest Control / Soil Fertility | 1.179575 | 0.503687 | 0.38677 | 1 | 0.38677 |
| Bird Conservation / Management of Captive Animals | 0.913098 | 0.217045 | -0.38246 | 1 | 0.382463 |
| Forest Conservation / Primate Conservation | 0.908508 | 0.228235 | -0.38194 | 1 | 0.381945 |
| Grassland Conservation / Primate Conservation | 1.119104 | 0.333097 | 0.378061 | 1 | 0.378061 |
| Farmland Conservation / Sustainable Aquaculture | 0.882107 | 0.325018 | -0.34045 | 1 | 0.340453 |
| Bird Conservation / Forest Conservation | 1.067599 | 0.20982 | 0.33283 | 1 | 0.33283 |
| Forest Conservation / Natural Pest Control | 1.129466 | 0.418309 | 0.328721 | 1 | 0.328721 |
| Bee Conservation / Subtidal Benthic Invertebrate Conservation | 0.90627 | 0.278533 | -0.32022 | 1 | 0.320224 |
| Management of Captive Animals / Marine Fish Conservation | 0.917263 | 0.254021 | -0.31185 | 1 | 0.311848 |
| Bat Conservation / Farmland Conservation | 1.075961 | 0.283205 | 0.278156 | 1 | 0.278156 |
| Natural Pest Control / Shrubland and Heathland Conservation | 0.901004 | 0.338958 | -0.2771 | 1 | 0.277102 |
| Bee Conservation / Biodiversity of Marine Artificial Structures | 0.904292 | 0.331197 | -0.27468 | 1 | 0.274683 |
| Sustainable Aquaculture / Terrestrial Mammal Conservation | 0.909133 | 0.316313 | -0.2738 | 1 | 0.273804 |
| Natural Pest Control / Reptile Conservation | 1.095078 | 0.369337 | 0.269295 | 1 | 0.269295 |
| Reptile Conservation / Soil Fertility | 1.077161 | 0.320338 | 0.249935 | 1 | 0.249935 |
| Grassland Conservation / Marsh and Swamp Conservation | 0.940207 | 0.246945 | -0.23474 | 1 | 0.234744 |
| Management of Captive Animals / Primate Conservation | 1.062232 | 0.302294 | 0.212143 | 1 | 0.212143 |
| Amphibian Conservation / Grassland Conservation | 1.057638 | 0.297334 | 0.19933 | 1 | 0.19933 |
| Bird Conservation / Primate Conservation | 0.969922 | 0.181585 | -0.16312 | 1 | 0.163125 |
| Grassland Conservation / Management of Captive Animals | 1.05354 | 0.349491 | 0.157224 | 1 | 0.157224 |
| Marine Fish Conservation / Marsh and Swamp Conservation | 0.972923 | 0.18365 | -0.14542 | 1 | 0.145422 |
| Bat Conservation / Sustainable Aquaculture | 0.949112 | 0.370912 | -0.13364 | 1 | 0.133644 |
| Grassland Conservation / Marine Fish Conservation | 0.966373 | 0.280577 | -0.11781 | 1 | 0.117812 |
| Bat Conservation / Peatland Conservation | 0.968258 | 0.265973 | -0.11743 | 1 | 0.117428 |
| Bat Conservation / Control of Freshwater Invasive Species | 0.967894 | 0.279353 | -0.11307 | 1 | 0.113066 |
| Amphibian Conservation / Marine Fish Conservation | 1.022072 | 0.218447 | 0.10215 | 1 | 0.10215 |
| Butterfly and Moth Conservation / Natural Pest Control | 0.970189 | 0.332612 | -0.08828 | 1 | 0.088277 |
| Forest Conservation / Shrubland and Heathland Conservation | 1.017653 | 0.27125 | 0.065653 | 1 | 0.065653 |
| Peatland Conservation / Sustainable Aquaculture | 0.980227 | 0.369294 | -0.05301 | 1 | 0.053011 |
| Control of Freshwater Invasive Species / Sustainable Aquaculture | 0.980596 | 0.379506 | -0.05063 | 1 | 0.050631 |
| Amphibian Conservation / Marsh and Swamp Conservation | 0.994398 | 0.173285 | -0.03224 | 1 | 0.032237 |
| Biodiversity of Marine Artificial Structures / Subtidal Benthic Invertebrate Conservation | 1.002188 | 0.323386 | 0.006772 | 1 | 0.006772 |
| Control of Freshwater Invasive Species / Peatland Conservation | 1.000377 | 0.269386 | 0.001398 | 1 | 0.001398 |

# Comparison of LLM performance with legacy model

Table S13 – Results of legacy LLM GPT-3.5 Instruct Turbo on a third of the unfiltered questions in terms of its accuracy at selecting the correct answer.

| Exam condition | Accuracy |
| --- | --- |
| Closed Book | 47.3% |
| Oracle | 77.5% |
| Confused | 73.5% |
| Dense | 62.3% |
| Sparse | 60.8% |
| Hybrid | 66.7% |
